# Phylogeny and Integrative Taxonomy of the Genera *Gymnaetoides* and *Pseudotachycines* (Orthoptera: Rhaphidophoridae) †

**DOI:** 10.3390/insects13070628

**Published:** 2022-07-14

**Authors:** Qidi Zhu, Haijian Wang, Zhijun Zhou, Fuming Shi

**Affiliations:** 1 Key Laboratory of Zoological Systematics and Application of Hebei Province, College of Life Sciences, Hebei University, Baoding 071002, China; qidizhu0323@163.com; 2 Institute of Life Sciences and Green Development, Hebei University, Baoding 071002, China; 3 College of Agronomy, Sichuan Agricultural University, Chengdu 611130, China; wanghaijian2005@sina.com

**Keywords:** Rhaphidophoridae, phylogeny, integrative taxonomy, new taxa

## Abstract

**Simple Summary:**

The genera *Gymnaetoides* and *Pseudotachycines* are morphologically homogeneous and species could not be classified according to the given generic diagnosis. Here, we reconstruct the phylogeny, and the results show that both *Gymnaetoides* and *Pseudotachycines* are paraphyletic. Therefore, we revise their taxonomy based on morphological characters and molecular data. A new genus is erected, and six new combinations and 15 new species are proposed. Moreover, we find that the shape of the male genitalia is important character for identifying these genera.

**Abstract:**

The genera *Gymnaetoides* and *Pseudotachycines* are endemic to China and are morphologically homogeneous. The few available diagnostic characters make species identification particularly challenging. Species cannot be classified according to the given generic diagnosis, and phylogenetic analyses have not been reported. Here, we reconstruct the phylogeny using Bayesian inference and maximum likelihood and employ four approaches to delimit species. The results suggest that both *Gymnaetoides* and *Pseudotachycines* are paraphyletic. Therefore, we revise their taxonomy based on the combination of morphological characters and molecular data. A new genus *Homotachycines* Zhu & Shi gen. nov. is erected, and six new combinations are proposed. Species delimitation identifies 15 new species and one new subspecies: *Gymnaetoides huangshanensis*, *G. petalus*, *G. yangmingensis*, *G. lushanensis*, *Pseudotachycines procerus*, *P. procerus guizhouensis*, *P. zhengi*, *P. nephrus*, *P. sagittus*, *P. fengyangshanensis*, *Homotachycines triangulus*, *H. quadratus*, *H. baokangensis*, *H. fusus*, *H. concavus*, and *H. qinlingensis* sp. nov. Moreover, we find that the shapes of the dorsal lateral lobes and the dorsal median lobe of the male genitalia are also important characters for identifying these genera and that the shapes of the dorsal and lateral sclerites of the male genitalia are suitable for the classifications of species.

## 1. Introduction

There are nine extant subfamilies and one extinct subfamily of Rhaphidophoridae, of which two subfamilies (Aemodogryllinae and Rhaphidophorinae) are recorded from China [1]. The subfamily Aemodogryllinae includes two tribes Aemodogryllini and Diestramimini, while Rhaphidophorinae includes one tribe Rhaphidophorini. Cave crickets prefer dark and moist habitats, most of them usually hide under litter or loose bark during the day and come out to hunt or mate at night, while others adapt to cave life [2,3,4]. They are wingless and do not have tibial tympana or a stridulatory organ. Moreover, their mating behavior is unique (the female on top). All of these are different from those of other families of Orthoptera. Despite the uniqueness of Rhaphidophoridae, they remain poorly studied. The monophyly of Rhaphidophoridae has been confirmed [5,6,7,8,9,10,11]. However, the phylogenetic relationship among the genera of Rhaphidophoridae has not been reported.

The tribe Aemodogryllini includes nine genera, of which the genera *Gymnaetoides*, *Pseudotachycines*, *Microtachycines,* and *Megatachycines* are characterized by the sclerotized dorsal lateral lobes of the male genitalia and are distinctly different from other genera [12,13,14]. The genus *Microtachycines* can easily be distinguished by the male tenth abdominal tergite, and *Megatachycines* is characterized by the male epiproct [15]. However, the genera *Gymnaetoides* and *Pseudotachycines* are morphologically homogeneous, which make species identification particularly challenging.

In the genus *Gymnaetoides*, *G. testaceus* Qin, Liu & Li, 2017 was assigned as the type species [13,16]. *Diestrammena* (*Gymnaeta*) *acutilobata* Gorochov, 2010 and *Diestrammena* (*Gymnaeta*) *indivisa* Zhang & Liu, 2009 were transferred to the genus *Gymnaetoides* according to the sclerotized apical area of the dorsal lateral lobes of the male genitalia and the normally developed male epiproct and paraproct [13,17,18,19]. Subsequently, one species from Guizhou was described, i.e., *Gymnaetoides polaoensis* Feng, Huang & Luo, 2020 [20]. However, according to the generic diagnosis and the characters, *G. polaoensis* may not belong to this genus.

For the genus *Pseudotachycines*, *P. deformis* Qin, Liu & Li, 2017 was assigned as the type species [14]. Five species were described, and *Diestrammena* (*Gymnaeta*) *ovalilobata* Gorochov, 2010 was transferred to it [14,17]. The genus *Pseudotachycines* was characterized by the elongated male paraproct and the sclerotized apical area of the dorsal lateral lobes of the male genitalia [14]. However, most species described in this paper did not provide morphological images of the male paraproct, and the line drawings of the male genitalia were too abstract to understand. Therefore, we examined the type specimens of these species at Shanghai Entomological Museum of the Chinese Academy of Sciences. We found that the line drawings of the male genitalia of these species did not adequately represent the characters and most species in the genus did not have the distinctly elongated male paraproct. Moreover, some species were more similar to the type species of the genus *Gymnaetoides*, such as *P. deformus* Qin, Liu & Li, 2017, *P. inermus* Qin, Liu & Li, 2017, and *P. yueyangensis* Qin, Liu & Li, 2017 [13,14]. Furthermore, due to few available diagnostic characters between them, species could not be classified according to the given generic diagnosis.

Here, we reconstruct the phylogeny of *Gymnaetoides* and *Pseudotachycines* to explore whether the genera are monophyletic. Moreover, we try to find more diagnostic characters for identifying the genera based on new molecular data. Last but not least, we would like to provide accurate morphological images for known species based on the examined type specimens.

## 2. Materials and Methods

### 2.1. Taxon Sampling

A total of 22 morphological species (subspecies) from Anhui, Chongqing, Guizhou, Henan, Hubei, Hunan, Jiangxi, Shaanxi, Yunnan, and Zhejiang were sampled (Table S1). Three specimens belonging to their relative genera *Tachycines* and *Diestrammena* were selected as outgroups. Species were identified under a Nikon SMZ800 stereomicroscope using original description [13,14,20]. The genitalia were dissected from the subgential plate with an insect needle and taken out with tweezer. Then, it was put in a 10% potassium hydroxide (KOH) solution to dissolve the soft tissues and transferred to glycerin for permanent preservation. Morphological images were acquired using a Leica DFC450 digital imaging system and edited using Adobe Photoshop CC 2018. Specimen measurements followed Zhu et al. [21].

Abbreviations for locations of specimens:

HBU—Museum of Hebei University, Baoding, China

SEMCAS—Shanghai Entomological Museum, Chinese Academy of Sciences, Shanghai, China.

### 2.2. DNA Extraction, Amplification, and Sequencing

Genomic DNA was extracted from muscle tissue of hind femur using TIANamp Genomic DNA Kit (Tiangen Biotech, Beijing, China). Extracted DNA was used as a template for the amplification of partial cytochrome c oxidase subunit I (*COI*), 12S rRNA, 16S rRNA, 18S rRNA, and 28S rRNA. The primers and polymerase chain reaction (PCR) amplifications followed that of Zhu et al. [21]. PCR products were examined on 1% agarose gel, and electrophoresis, purification, and bi-directional sequencing were performed at GENEWIZ (Tianjin, China).

### 2.3. Sequence Alignment and Phylogenetic Analyses

Sequences were edited using SeqMan in DNASTAR’s Lasergene v.7.1 and aligned in MAFFT v.7.313 [22,23]. For *COI* sequences, in order to minimize the possibility of co-amplification of nuclear mitochondrial pseudogenes (Numts) [24,25,26], we translated them into amino acid sequences to check for stop codons using EditSeq in DNASTAR’s Lasergene v.7.1 and refined them using MACSE v.2.03 [22,27]. Then, the alignments were manually optimized and concatenated into a dataset using SequenceMatrix v.1.8 [28]. The final matrix contained 2803 bp (681 bp of *COI*, 391 bp of 12S rRNA, 522 bp of 16S rRNA, 572 bp of 18S rRNA, and 637 bp of 28S rRNA). All sequences were deposited in GenBank (accession numbers are reported in Table S1).

Nucleotide substitution models were estimated using ModelFinder based on the Bayesian information criterion (BIC) with edge-linked features [29]. *COI* was partitioned by codon positions. The best-fitting models were as follows: SYM + G for *COI*_pos1, F81 for *COI*_pos2, HKY + G for *COI*_pos3, GTR + G + I for 12S rRNA + 16S rRNA, and JC + G for 18S rRNA + 28S rRNA.

Phylogenetic analyses were conducted using Bayesian inference (BI) and maximum likelihood (ML) approaches. The ML analysis was performed using IQ-TREE v.1.6.8 [30] with 5000 ultrafast bootstraps [31] in Phylosuit v.1.2.2 [32]. Bootstrap support values (BS) above 70% were considered to be well supported [33]. The BI analysis was performed using MrBayes v.3.2.6 [34] in Phylosuit v.1.2.2 [32] with two independent runs and four Markov Chain Monte Carlo (MCMC) chains. The analysis was run for 10^6^ generations with sampling every 100 generations, and the initial 25% of sampled data were discarded as burn-in, whereas the others were used to calculate the posterior probabilities (PP) values. PPs above 95% were considered to be strongly supported [35]. The convergence of the runs was assessed by examining the average standard deviation of split frequencies (ASDSF) and effective sample sizes (ESS) of all parameters using Tracer 1.7.1 [36]. A value of ESS > 200 was considered a good indicator of convergence. The obtained dendrograms were visualized and edited with FigTree 1.4.4 [37].

### 2.4. Species Delimitation

Four species delimitation approaches were employed to explore species boundaries based on the *COI* dataset. Automatic barcode gap discovery (ABGD; [38]) and java molecular operational taxonomic units (jMOTU; [39]) are distance-based methods. ABGD was conducted based on the Kimura (K80) TS/TV 2.0 model with prior value of intraspecific divergence between 0.001 and 0.1, 10 recursive steps, and relative gap width (X) of 0.5. For jMOTU analysis, the low blast identity filter was set to 97% and the percentage of minimum sequence length was set to 95. The general mixed yule coalescent model (GMYC; [40,41]) and the Bayesian implementation of the Poisson tree process model (bPTP; [42]) are tree-based methods. The GMYC analysis was implemented based on ultrametric trees, which were generated in Beast v.1.10.4 [43]. The best partitioning scheme was estimated using ModelFinder [29]. A lognormal relaxed clock and a birth–death tree prior were implemented as the tree prior [44,45]. The MCMC was run for 10^7^ generations with sampling for every 1000 generations. The ESSs were greater than 200 for all parameters. The maximum clade credibility tree with a 10% burn-in was generated in TreeAnnotator v.1.10.4 [43]. The single-threshold method was used to generate the putative species. The bPTP analysis was performed based on the BI tree generated from Mrbayes 3.2.6 [34], and MOTUs were calculated using Bayesian and ML methods.

## 3. Results

### 3.1. Phylogenetic Analyses

**Figure 1:**
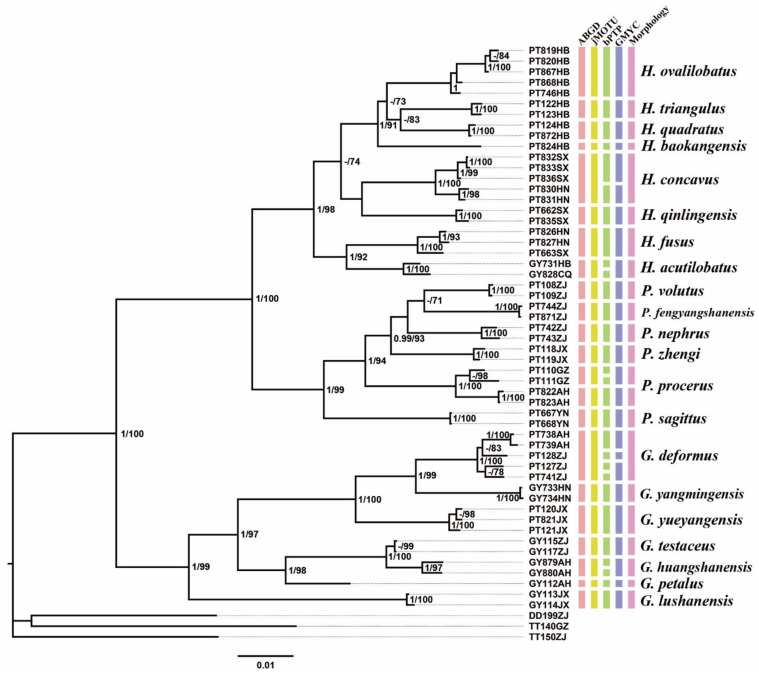
BI tree of *Gymnaetoides*, *Pseudotachycines,* and *Homotachycines*. The numbers at the nodes are Bayesian posterior probabilities (PP) and maximum likelihood bootstrap support values (BS). PP > 0.95 or BS > 70 are given at the nodes. In ML tree, PT868HB is recovered as sister to PT746HB (BS = 92) and together they are sister to PT819HB, PT820HB, and PT867HB (BS = 100). Results of molecular species delimitation based on ABGD, jMOTU, bPTP, GMYC, and morphological species are annotated on the right side of the tree.

The topologies of BI and ML trees were largely congruent, and the supporting values or posterior probabilities were high (Figure 1). Phylogenetic analyses recovered three main clades, which were inconsistent with the current classification scheme of *Gymnaetoides* and *Pseudotachycines*. *P. deformis* and *P. yueyangensis* were clustered into the clade *Gymnaetoides*. *P. volutus* formed a separate clade, while *P. ovalilobatus* and *G. acutilobatus* were clustered into a clade. Both *Gymnaetoides* and *Pseudotachycines* were paraphyletic.

According to the given generic diagnosis, *Gymnaetoides* is characterized by the distinctly sclerotized apex of the dorsolateral lobes of the male genitalia and the normally developed male epiproct and paraproct, while *Pseudotachycines* is characterized by the sclerotized apex of the dorsolateral lobes of the male genitalia and the distinctly prolonged male paraproct. Therefore, the clade *Gymnaetoides* contains seven species and *P. deformis* and *P. yueyangensis* should be transferred to it. In the clade *Pseudotachycines*, only the known species *P. volutus* and the five new species are included. The third clade represents a new taxon and thus a new genus *Homotachycines* Zhu & Shi gen. nov. is proposed. *Homotachycines* is sister to *Pseudotachycines*, and they are together sisters to *Gymnaetoides*. Moreover, *P. ovalilobatus* and *G. acutilobatus* should be transferred to *Homotachycines*.

### 3.2. Species Delimitation

The results of the four species delimitation methods were inconsistent (Figure 1). Although the ABGD and jMOTU analyses were consistent with the morphological species in clustering 22 MOTUs, the bPTP analysis generated 29 MOTUs and the GMYC analysis resulted in 25 MOTUs. Moreover, there was also inconsistency between molecular data and morphological species. In *Pseudotachycines procerus*, specimens from Guizhou and Anhui formed two MOTUs in all of the four species delimitation methods. Despite the inconsistency of different species delimitation methods or the inconsistency of morphological and genetic data, most species (17 of 22) can successfully be identified.

### 3.3. Taxonomy

**Key to genera *Gymnaetoides*, *Pseudotachycines,* and *Homotachycines***

1. The apical area of the dorsal median lobe of the male genitalia divides into two lobes; the dorsal lateral lobe of the male genitalia is of nearly equal length as the dorsal median lobe, in which the apical area is distinctly sclerotized………...***Gymnaetoides***

- The apical area of the dorsal median lobe of the male genitalia does not divide; the dorsal lateral lobe of the male genitalia is much or little longer than the dorsal median lobe, in which the apical area is weakly sclerotized…………………………….……...2

2. The male paraproct is prolonged; the dorsal lateral lobe of the male genitalia is much longer than the dorsal median lobe, in which the apical area is almost as wide as the basal area………………………………………..………………...…...***Pseudotachycines***

- The male paraproct is normally developed; the dorsal lateral lobe of the male genitalia is little longer than the dorsal median lobe, in which the apical area is much narrower than the basal area……………………………………………... ***Homotachycines***

**Genus *Gymnaetoides* Qin, Liu & Li, 2017**

*Gymnaetoides* Qin, Liu & Li, 2017: 186; Feng, Huang & Luo, 2020: 447.

**Type species.** *Gymnaetoides testaceus* Qin, Liu & Li, 2017, by original designation.

**Diagnosis.** Body is smaller than other genera in Aemodogryllini. All of the femora are unarmed on ventral surfaces or only the hind femur with one inner spine on ventral surface. The male epiproct and paraproct are normally developed. The male genitalia has an unpaired dorsal sclerite; the dorsal median lobe of the male genitalia has paired lateral sclerites at the basal area, and the apical area divides into two lobes; the dorsal lateral lobe of the male genitalia is broad, nearly equal in length to dorsal median lobe, and the apical area is sclerotized.

***Gymnaetoides testaceus* Qin, Liu & Li, 2017**

*Gymnaetoides testaceus* Qin, Liu & Li, 2017: 188.

(Figure 2A)

**Material examined.** Holotype: ‘3♂1♀’ (Qin et al., 2017a), China, Zhejiang, Kaihua, Gutian Mountain, 14~16.Ⅹ.2012, coll. X.W. Liu and H.Q. Wang, dep. SEMCAS. Paratypes: 1♂1♀, same data as for holotype. Other material: 13♂11♀, China, Zhejiang, Kaihua, Gutian Mountain, 8.Ⅹ.2018, coll. T. Wang, dep. HBU; 3♂17♀, China, Zhejiang, Kaihua, Gutian Mountain, 10.Ⅹ.2018, coll. T. Wang, dep. HBU; 1♂, China, Zhejiang, Linan, Tianmu Mountain, 12.Ⅹ.2018, coll. T. Wang, dep. HBU.

**Distribution.** China (Zhejiang).

**Figure 2:**
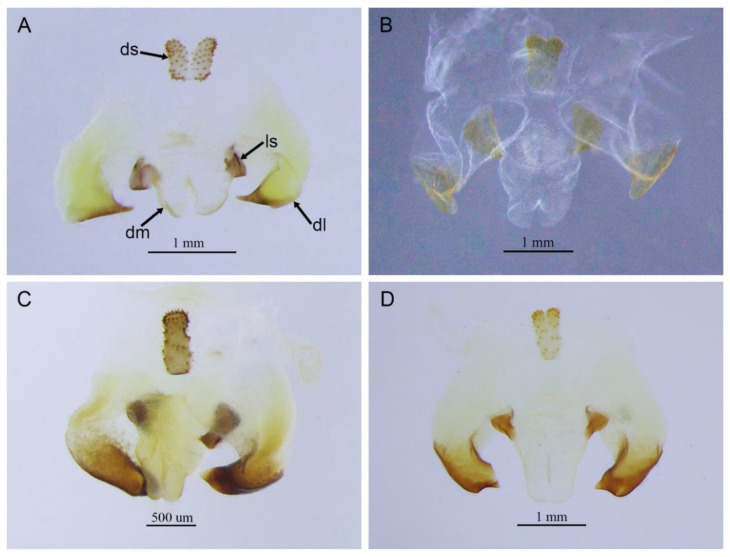
Male genitalia of *Gymnaetoides* (type specimens). (**A**) *Gymnaetoides testaceus*; (**B**) *Gymnaetoides deformus* comb. nov.; (**C**) *Gymnaetoides inermus* comb. nov.; (**D**) *Gymnaetoides yueyangensis* comb. nov. Abbreviations: ds = dorsal sclerite; ls = lateral sclerite; dm = dorsal median lobe; dl = dorsal lateral lobe.

**Figure 3:**
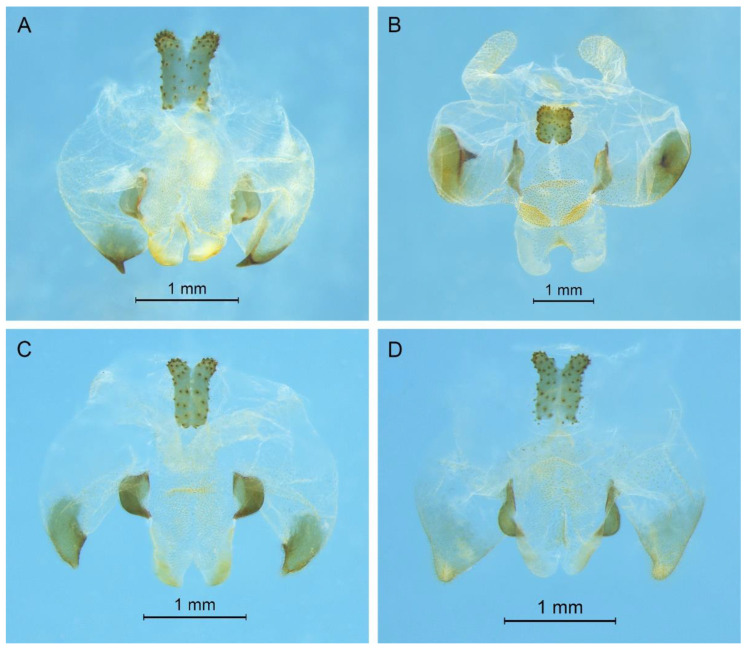
Male genitalia of *Gymnaetoides*. (**A**) *Gymnaetoides huangshanensis* Zhu & Shi sp. nov.; (**B**) *Gymnaetoides petalus* Zhu & Shi sp. nov.; (**C**) *Gymnaetoides yangmingensis* Zhu & Shi sp. nov.; (**D**) *Gymnaetoides lushanensis* Zhu & Shi sp. nov.

**Remarks.** The species was recorded in Zhejiang (Gutian Mountain, Tianmu Mountain) and Anhui (Huang Mountain). In the original description, 65–68 inner and 64–69 outer spines on dorsal surface of the hind tibia were reported. However, after examining the type and topotype specimens, 30–39 inner and 37–40 outer spines on dorsal surface of the hind tibia in specimens from Zhejiang were observed, while 60–69 inner spines and 64–69 outer spines were observed in specimens collected from Anhui. This was also indicated by Qin [16]. Moreover, all of the four species delimitation approaches suggest that the specimens from Zhejiang and Anhui are two separate species (Figure 1). Therefore, we consider the population from Anhui as a new species, *Gymnaetoides huangshanensis* Zhu & Shi sp. nov. (described below). *G. testaceus* is currently found only in Zhejiang and the description of spines on hind tibia should be changed to “hind tibia with 30–39 inner spines and 37–40 outer spines on dorsal surface”. In addition, the line drawings of the male genitalia did not adequately represent the characters (Figures 3 and 4 in [13]). The photograph of type specimen is shown in Figure 2A.

**Figure 4:**
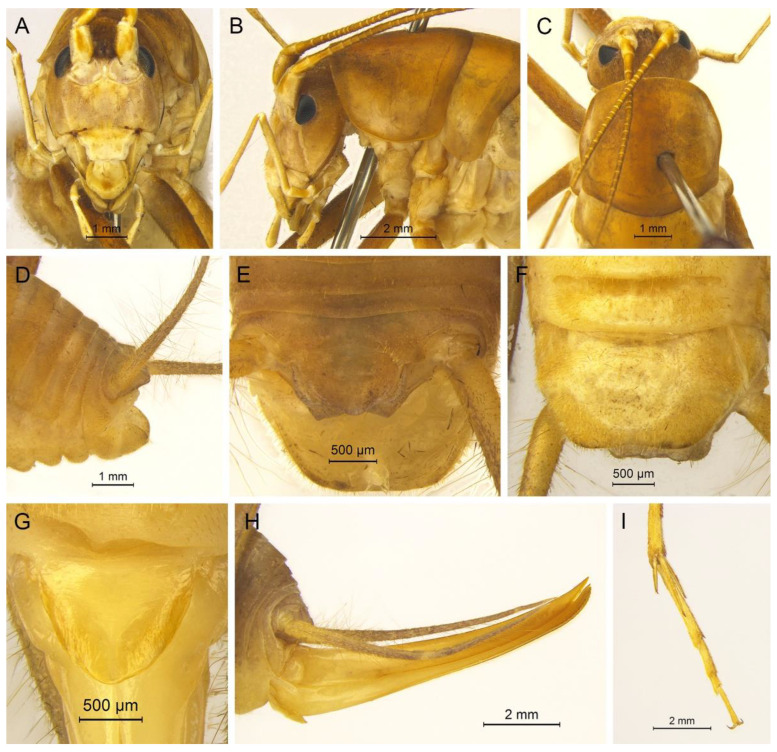
*Gymnaetoides huangshanensis* Zhu & Shi sp. nov., male: (**A**–**C**) head and pronotum: (**A**) frontal view, (**B**) lateral view, (**C**) dorsal view; (**D**–**F**) apex of abdomen: (**D**) lateral view, (**E**) dorsal view, (**F**) ventral view; (**I**) hind tarsus in lateral view; female: (**G**) subgenital plate in ventral view; (**H**) ovipositor in lateral view.

***Gymnaetoides huangshanensis* Zhu & Shi sp. nov.**

urn:lsid:zoobank.org:act:A80F697E-9710-4268-839E-44603F1B7D5B

(Figure 3A and Figure 4)

**Material examined.** Holotype: ♂, China, Anhui, Huang Mountain, Tangkou, 15.Ⅸ.2019, coll. T. Wang, dep. HBU. Paratypes: 3♂4♀, same data as for holotype. Other material: 3♂, same data as for holotype.

**Etymology.** The name of the new species derives from the type locality.

**Diagnosis.** The new species is similar to *G. testaceus* Qin, Liu & Li, 2017, but it can be distinguished by the shape of the male genitalia and by more dorsal spines on the hind tibia. The number of dorsal spines on the hind tibia of *G. testaceus* is 30–39 inner spines and 37–40 outer spines, while that of *G. huangshanensis* is 60–69 inner spines and 64–69 outer spines. The dorsal sclerite of the male genitalia is quadrangular, the basal area broad with concavity, the apical area slightly concave; lateral sclerites of dorsal median lobe are nearly semicircular.

**Description.** Male: Body medium sized in *Gymnaetoides*. Fastigium verticis with two conical tubercles, apices rounded, directing forward. Eyes ovoid, protruding forward; median ocellus oval, located between antennal sockets; lateral ocelli circular, situated on lateral margins of basal fastigium verticis. Apical segment of maxillary palpus much longer than subapical segment, apex inflated, globular.

Pronotum long, anterior margin of disc straight, posterior margin arcuate; lateral lobes longer than high, ventral margins arc shaped. Mesonotum and metanotum short, posterior margin of mesonotum arcuate, posterior margin of metanotum straight. Fore coxa with one small spine; femur unarmed on ventral surface, internal genicular lobe with one small spine, external genicular lobe with one long spine; tibia with one inner spine on ventral surface, apical area with one extero-dorsal spine and a pair of ventral spines, between the paired ventral spines with one small spine. Mid femur unarmed on ventral surface, internal and external genicular lobes each with one long spine; tibia with one inner spine and one outer spine on ventral surface, apical area with a pair of dorsal spines and a pair of ventral spines, between the paired ventral spines with one small spine. Hind femur unarmed on ventral surface, internal and external genicular lobes unarmed; tibia with 60–69 inner spines and 64–68 outer spines on dorsal surface, with a pair of dorsal spines in subapical area, and at apex with a pair of dorsal spines and two pairs of ventral spines, intero-dorsal spine shorter than hind basitarsus. Hind basitarsus with one dorsal apical spine.

Posterior margins of all abdominal tergites straight. Epiproct ligulate, paraproct triangular in lateral view. Cercus slender, conical, apex acute. Dorsal sclerite of genitalia quadrangular, basal area broad, with concavity, apical area slightly concave; basal area of dorsal median lobe with paired lateral sclerites, nearly semicircular, apical area divided into two lobes; dorsal lateral lobes broad, nearly equal in length to dorsal median lobe, apical area sclerotized, spinous; ventral lateral lobes tiny, much shorter than dorsal lateral lobes; ventral median lobe short. Subgenital plate quadrangular, transverse and wide, posterior margin slightly concave.

Female: Appearance is similar to male. Ovipositor longer than half the length of hind femur, slightly curved upward, dorsal margin smooth, apical area of ventral margin denticulate. Subgenital plate triangular, apex obtuse.

**Coloration.** Body yellowish brown, uniform. Eyes black, ocelli yellow.

**Measurements (mm).** Body: ♂10.9–13.8, ♀9.5–11.6; pronotum: ♂4.1–4.5, ♀3.5–3.8; fore femur: ♂9.3–10.0, ♀8.0–8.4; hind femur: ♂16.5–18.6, ♀13.8–14.6; hind tibia: ♂17.7–19.2, ♀14.8–16.0; hind basitarsus: ♂4.3–4.4, ♀3.3–3.7; ovipositor: 8.6–9.5.

**Distribution.** China (Anhui).

***Gymnaetoides indivisus* (Zhang & Liu, 2009)**

*Diestrammena* (*Gymnaeta*) indivisa Zhang & Liu, 2009: 26.

*Gymnaetoides indivisus*: Qin, Liu & Li, 2017: 188.

**Material examined.** Holotype: ♂, China, Jiangxi, Wannian County, 6.Ⅸ.2007, coll. Jiao et al., dep. SEMCAS. Paratype: 1♂, same data as for holotype.

**Distribution.** China (Jiangxi).

**Remarks.** The characters of this species were very similar to those of *G. testaceus* and thus it was regarded as a synonym of *G. testaceus* [16]. However, the line drawings of *G. indivisus* were too abstract and the type specimen was not well preserved. It is difficult to judge whether they are the same species. Therefore, we regard it as a suspect species temporarily, and more evidence is needed for further study.

***Gymnaetoides deformus* (Qin, Liu & Li, 2017) comb. nov.**

*Pseudotachycines deformis* Qin, Liu & Li, 2017: 483.

(Figure 2B)

**Material examined.** Holotype: ♂, China, Zhejiang, Linan, Tianmu Mountain, 14~15.Ⅹ.2009, coll. X.W. Liu, dep. SEMCAS. Paratypes: 9♂16♀, same data as for holotype. Other material: 1♂2♀, China, Anhui, Huang Mountain, Tangkou, 15.Ⅸ.2019, coll. Y.Q. Li, dep. HBU; 5♂3♀, China, Zhejiang, Linan, Qiangliangfeng, 4.Ⅹ.2019, coll. Q.D. Zhu, dep. HBU; 5♂4♀, China, Zhejiang, Linan, Tianmu Mountain, 30.Ⅸ.2019, coll. Q.D. Zhu, dep. HBU; 4♂8♀, China, Zhejiang, Linan, Tianmu Mountain, 11.Ⅹ.2018, coll. T. Wang, dep. HBU; 2♂13♀, Tianmu Mountain, Linan, Zhejiang, China, 12.Ⅹ.2018, coll. T. Wang, dep. HBU.

**Distribution.** China (Anhui, Zhejiang).

**Remarks.** This species was designated as the type species of *Pseudotachycines*, which was characterized by the prolonged male paraproct [14]. However, after examining the type specimen, we did not find the specialized paraproct. Moreover, the line drawings of the male genitalia did not adequately represent the characters (Figures 4 and 5 in [14]). The photograph of the type specimen is shown in Figure 2B. The result of phylogenetic analyses also clusters this species in genus *Gymnaetoides* (Figure 1). It should be transferred into the genus *Gymnaetoides* according to the characters of the male genitalia: the apical area of the dorsal median lobe divided into two lobes, the dorsal lateral lobes broad, nearly equal in length to dorsal median lobe, the apical area sclerotized.

***Gymnaetoides inermus* (Qin, Liu & Li, 2017) comb. nov.**

*Pseudotachycines inermis* Qin, Liu & Li, 2017: 483.

(Figure 2C)

**Material examined.** Holotype: ♂, China, Hubei, Xianning, Jiugong Mountain, 30.Ⅶ.2016, coll. Hu and Liu, dep. SEMCAS.

**Distribution.** China (Hubei).

**Remarks.** The species was classified into *Pseudotachycines* based on the prolonged male paraproct [14]. However, after examining the type specimen, this feature was not distinct. The line drawings of the male genitalia did not adequately represent the characters (Figures 8 and 9 in [14]). The photograph of the type specimen is shown in Figure 2C. The dorsal sclerite of the male genitalia is rectangular, much longer than wide; the lateral sclerite of the dorsal median lobe is nearly rectangular, with the apical area divided into two lobes; the dorsal lateral lobe is broad, nearly equal in length to the dorsal median lobe, with the apical area sclerotized; the ventral lateral lobe and the ventral median lobe are short. According to the characters of the male genitalia, it should be transferred into the genus *Gymnaetoides*.

***Gymnaetoides yueyangensis* (Qin, Liu & Li, 2017) comb. nov.**

Pseudotachycines yueyangensis Qin, Liu & Li, 2017: 485.

(Figure 2D)

**Material examined.** Holotype: ♂, China, Hunan, Yueyang, Forest Park of Fushou Mountain, 18~26.Ⅹ.2016, coll. Jiang et al., dep. SEMCAS. Paratypes: 6♂6♀, same data as for holotype. Other material: 6♂3♀, China, Jiangxi, Lu Mountain, Guling, 7.Ⅷ.2018, coll. T. Wang, dep. HBU.

**Distribution.** China (Hunan, Jiangxi).

**Remarks.** After examining the type specimen, we found that the line drawings of the male genitalia did not adequately represent the characters (Figures 10 and 11 in [14]). The photograph of the type specimen is shown in Figure 2D. The apical area of the dorsal median lobe of the male genitalia divides into two lobes; the dorsal lateral lobe is broad, nearly equal in length to the dorsal median lobe, with the apical area sclerotized. According to the characters of the male genitalia and the phylogenetic analyses, we transfer it into genus *Gymnaetoides*.

**Figure 5:**
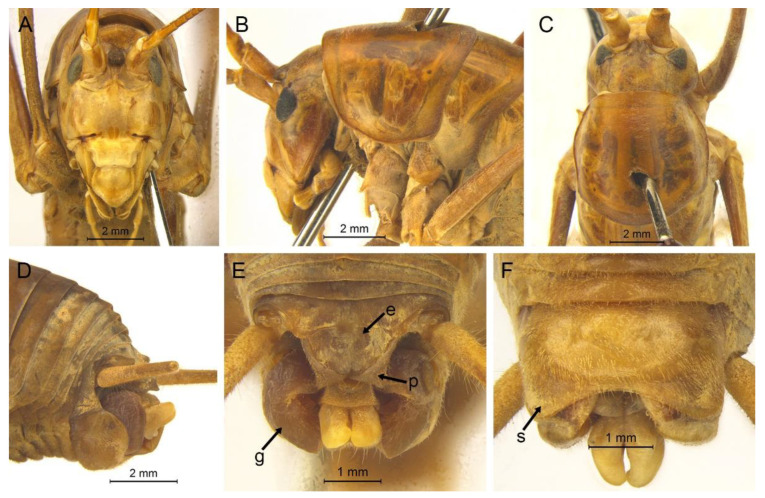
*Gymnaetoides petalus* Zhu & Shi sp. nov., male: (**A**–**C**) head and pronotum: (**A**) frontal view, (**B**) lateral view, (**C**) dorsal view; (**D**–**F**) apex of abdomen: (**D**) lateral view, (**E**) dorsal view, (**F**) ventral view. Abbreviations: e = epiproct; p = paraproct; g = genitalia; s = subgenital plate.

***Gymnaetoides petalus* Zhu & Shi sp. nov.**

urn:lsid:zoobank.org:act:6395D039-02AF-464D-A89A-5B33A3E7DBD0

(Figure 3B and Figure 5)

**Material examined.** Holotype: ♂, China, Anhui, Chizhou City, Shitai County, Gongxi Village, Du hamlet, Penglaixian Cave, 17.Ⅶ.2014, coll. M.Y. Tian, dep. HBU. Paratype: 1♂, same data as for holotype.

**Etymology.** The name of the new species derives from the Greek word ‘*petal*’ (petaloid), referring to the petaloid dorsal sclerite of the male genitalia.

**Diagnosis.** The new species can easily be distinguished from known congeneric species by the shape of the male genitalia: the dorsal sclerite of the male genitalia is petaloid and the lateral sclerite of the dorsal median lobe is slender.

**Description.** Male: Body medium sized in *Gymnaetoides*. Fastigium verticis with two conical tubercles, apices divided, directing forward. Eyes ovoid, protruding forward; median ocellus oval, located between antennal sockets; lateral ocelli circular, situated on lateral margins of basal fastigium verticis. Apical segment of maxillary palpus little longer than subapical segment, apex truncate.

Anterior margin of pronotum straight, posterior margin arcuate; lateral lobes longer than high, ventral margins arc shaped. Mesonotum and metanotum short, posterior margin of mesonotum arcuate, posterior margin of metanotum straight. Fore coxa with one small spine; femur unarmed on ventral surface, internal genicular lobe with one small spine, external genicular lobe with one long spine; tibia with two outer spines on ventral surface, apical area with one extero-dorsal spine and a pair of ventral spines, between the paired ventral spines with one small spine. Mid femur unarmed on ventral surface, internal and external genicular lobes each with one long spine; tibia with one inner spine and one outer spine on ventral surface, apical area with a pair of dorsal spines and a pair of ventral spines, between the paired ventral spines with one small spine. Hind femur unarmed on ventral surface; tibia with 87–92 inner spines and 84–86 outer spines on dorsal surface, with a pair of dorsal spines in subapical area, and at apex with a pair of dorsal spines and two pairs of ventral spines, intero-dorsal spine shorter than hind basitarsus. Hind basitarsus with one dorsal apical spine.

All abdominal tergites without process. Epiproct ligulate, paraproct slightly longer than epiproct. Cercus slender, conical, apex acute. Dorsal sclerite of genitalia petaloid; basal area of dorsal median lobe with paired lateral sclerites, slender, apical area divided into two lobes; dorsal lateral lobes broad, nearly equal in length to dorsal median lobe, apical area sclerotized, spinous; ventral lateral lobes cylindrical, much shorter than dorsal lateral lobes; ventral median lobe short. Subgenital plate transverse and wide, posterior margin widely concave.

Female: Unknown.

**Coloration.** Body yellowish brown, uniform.

**Measurements (mm).** Body: ♂12.6–13.9; pronotum: ♂6.6–6.8; fore femur: ♂9.7–11.8; hind femur: ♂18.2–18.4; hind tibia: ♂19.9–20.4; hind basitarsus: ♂4.4–4.9.

**Distribution.** China (Anhui).

**Figure 6:**
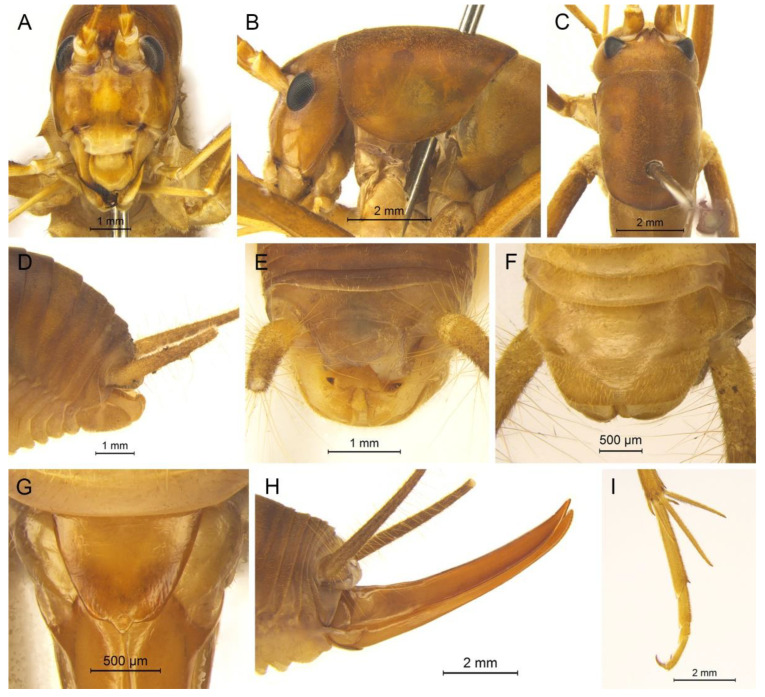
*Gymnaetoides yangmingensis* Zhu & Shi sp. nov., male: (**A**–**C**) head and pronotum: (**A**) frontal view, (**B**) lateral view, (**C**) dorsal view; (**D**–**F**) apex of abdomen: (**D**) lateral view, (**E**) dorsal view, (**F**) ventral view; (**I**) hind tarsus in lateral view; female: (**G**) subgenital plate in ventral view; (**H**) ovipositor in lateral view.

***Gymnaetoides yangmingensis* Zhu & Shi sp. nov.**

urn:lsid:zoobank.org:act:AEFABE7E-E962-47A7-B2EB-37CF8E3D6EE4

(Figure 3C and Figure 6)

**Material examined.** Holotype: ♂, China, Hunan, Shuangpai, Yangming Mountain, 21.Ⅷ.2019, coll. Y.R. Xin, dep. HBU. Paratypes: 2♂4♀, same data as for holotype. Other material: 1♂8♀, same data as for holotype.

**Etymology.** The name of the new species refers to the type locality.

**Diagnosis.** The new species is similar to *Gymnaetoides yueyangensis* (Qin, Liu & Li, 2017), but it differs from the latter one by the shape of the male genitalia. The dorsal sclerite of the male genitalia is quadrangular, with a concavity on the basal area; the lateral sclerite of the dorsal median lobe is semicircular; the apical area of the dorsal lateral lobe is spinous.

**Description.** Male: Body medium sized in *Gymnaetoides*. Fastigium verticis with two conical tubercles, apices divided, directing forward. Eyes ovoid, protruding forward; median ocellus oval, located between antennal sockets; lateral ocelli circular, situated on lateral margins of basal fastigium verticis. Apical segment of maxillary palpus little longer than subapical segment, apex inflated, globular.

Pronotum long, anterior margin of disc straight, posterior margin arcuate; lateral lobes longer than high, ventral margins arc shaped. Mesonotum and metanotum short, posterior margin of mesonotum arcuate, posterior margin of metanotum straight. Fore coxa with one small spine; femur unarmed on ventral surface, internal genicular lobe with one small spine, external genicular lobe with one long spine; tibia with two outer spines on ventral surface, apical area with one extero-dorsal spine and a pair of ventral spines, between the paired ventral spines with one small spine. Mid femur unarmed on ventral surface, internal and external genicular lobes each with one long spine; tibia with one inner spine and one outer spine on ventral surface, apical area with a pair of dorsal spines and a pair of ventral spines, between the paired ventral spines with one small spine. Hind femur unarmed or with one spine on ventral surface, internal genicular lobe with one small spine; tibia with 50–56 inner spines and 51–53 outer spines on dorsal surface, with a pair of dorsal spines in subapical area, and at apex with a pair of dorsal spines and two pairs of ventral spines, intero-dorsal spine nearly equal in length to hind basitarsus. Hind basitarsus with one dorsal apical spine.

All abdominal tergites without process. Epiproct ligulate, paraproct triangular in lateral view. Cercus slender, conical, apex acute. Dorsal sclerite of genitalia quadrangular, basal area wide, with concavity; basal area of dorsal median lobe with paired lateral sclerites, semicircular, apical area divided into two lobes; dorsal lateral lobes broad, nearly equal in length to dorsal median lobe, apical area spinous; ventral lateral lobes much shorter than dorsal lateral lobes; ventral median lobe short. Subgenital plate transverse and wide, posterior margin straight.

Female: Appearance is similar to male. Ovipositor longer than half the length of hind femur, slightly curved upward, dorsal margin smooth, apical area of ventral margin denticulate. Subgenital plate trapezoidal, posterior margin slightly protruding.

**Coloration.** Body light brown, uniform.

**Measurements (mm).** Body: ♂10.3–11.7, ♀11.9–12.7; pronotum: ♂4.2–4.5, ♀3.8–4.6; fore femur: ♂6.3–7.4, ♀7.1–7.6; hind femur: ♂14.1–14.7, ♀14.7–15.4; hind tibia: ♂14.3–15.2, ♀15.4–16.4; hind basitarsus: ♂3.3–4.1, ♀3.3–3.6; ovipositor: 8.1–8.3.

**Distribution.** China (Hunan).

**Figure 7:**
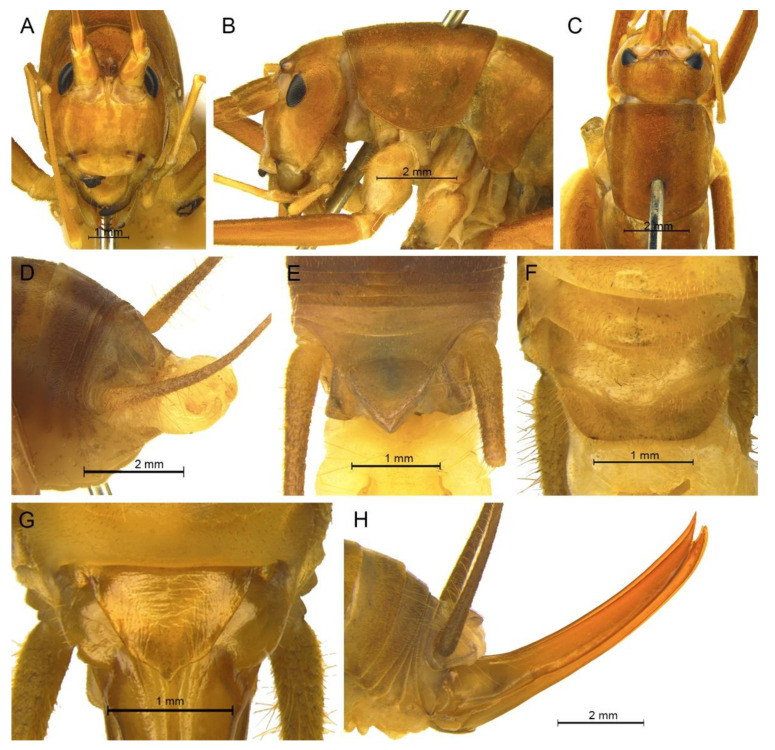
*Gymnaetoides lushanensis* Zhu & Shi sp. nov., male: (**A**–**C**) head and pronotum: (**A**) frontal view, (**B**) lateral view, (**C**) dorsal view; (**D**–**F**) apex of abdomen: (**D**) lateral view, (**E**) dorsal view, (**F**) ventral view; female: (**G**) subgenital plate in ventral view; (**H**) ovipositor in lateral view.

***Gymnaetoides lushanensis* Zhu & Shi sp. nov.**

urn:lsid:zoobank.org:act:0DC9C41D-1105-4D8A-882D-FBFA70F04889

(Figure 3D and Figure 7)

**Material examined.** Holotype: ♂, China, Jiangxi, Lu Mountain, Guling, 7.Ⅷ.2018, coll. T. Wang, dep. HBU. Paratypes: 1♀, same data as for holotype; 1♂1♀, China, Jiangxi, Lu Mountain, Guling, 6.Ⅷ.2018, coll. T. Wang, dep. HBU. Other material: 2♂, China, Jiangxi, Lu Mountain, Dayueshan, 3.Ⅷ.2018, coll. T. Wang, dep. HBU; 2♂2♀, China, Jiangxi, Lu Mountain, Guling, 6.Ⅷ.2018, coll. T. Wang, dep. HBU; 5♂2♀, China, Jiangxi, Lu Mountain, Guling, 7.Ⅷ.2018, coll. T. Wang, dep. HBU.

**Etymology.** The name of the new species derives from the type locality.

**Diagnosis.** The new species is similar to *Gymnaetoides huangshanensis* Zhu & Shi sp. nov., but it can be distinguished by the shapes of lateral sclerite of the dorsal median lobe and the dorsal lateral lobe of the male genitalia. The lateral sclerite of the dorsal median lobe is spoon shaped and the apical area of the dorsal lateral lobe is slightly sclerotized.

**Description.** Male: Body medium sized in *Gymnaetoides*. Fastigium verticis with two conical tubercles, apices divided, directing forward. Eyes ovoid, protruding forward; median ocellus oval, located between antennal sockets; lateral ocelli circular, situated on lateral margins of basal fastigium verticis. Apical segment of maxillary palpus much longer than subapical segment, apex inflated, globular.

Pronotum long, anterior margin of disc straight, posterior margin arcuate; lateral lobes longer than high, ventral margins arc shaped. Mesonotum and metanotum short, posterior margin of mesonotum arcuate, posterior margin of metanotum straight. Fore coxa with one small spine; femur unarmed on ventral surface, internal genicular lobe with one small spine, external genicular lobe with one long spine; tibia with two outer spines on ventral surface, apical area with one extero-dorsal spine and a pair of ventral spines, between the paired ventral spines with one small spine. Mid femur unarmed on ventral surface, internal and external genicular lobes each with one long spine; tibia with one inner spine and two outer spines on ventral surface, apical area with a pair of dorsal spines and a pair of ventral spines, between the paired ventral spines with one small spine. Hind femur unarmed on ventral surface; tibia with 65–75 inner spines and 63–71 outer spines on dorsal surface, with a pair of dorsal spines in subapical area, and at apex with a pair of dorsal spines and two pairs of ventral spines, intero-dorsal spine slightly shorter than hind basitarsus. Hind basitarsus with one dorsal apical spine.

Posterior margins of all abdominal tergites straight. Epiproct ligulate, paraproct simple, triangular in lateral view. Cercus slender, conical, apex acute. Dorsal sclerite of genitalia quadrangular, basal area wide, with a concavity, apex slightly concave; basal area of dorsal median lobe with paired lateral sclerites, spoon-shaped, apical area divided into two lobes; dorsal lateral lobes broad, nearly equal in length to dorsal median lobe, apical area slightly sclerotized; ventral lateral lobes cylindrical, nearly equal in length to dorsal lateral lobes; ventral median lobe short. Subgenital plate transverse and wide, posterior margin straight.

Female: Appearance is similar to male. Ovipositor longer than half the length of hind femur, dorsal margin smooth, apical area of ventral margin denticulate. Subgenital plate triangular, apex acute.

**Coloration.** Body yellow. Pronotum and apex of abdomen yellowish brown.

**Measurements (mm).** Body: ♂10.0–11.5, ♀11.0–12.5; pronotum: ♂4.2–5.5, ♀4.5–5.4; fore femur: ♂9.0–9.5, ♀7.0–8.0; hind femur: ♂15.2–16.0, ♀14.5–15.8; hind tibia: ♂15.2–16.3, ♀15.5–17.0; hind basitarsus: ♂4.0–4.4, ♀3.5–4.2; ovipositor: 8.5–9.0.

**Distribution.** China (Jiangxi).

**Genus *Pseudotachycines* Qin, Liu & Li, 2017**

*Pseudotachycines* Qin, Liu & Li, 2017: 482.

**Type species.** *Pseudotachycines volutus* Qin, Liu & Li, 2017, here redesignation.

**Diagnosis.** Body is medium sized in Aemodogryllini. The male paraproct are specialized, slightly or much longer than epiproct. The male genitalia have unpaired dorsal sclerite, smaller than that of the other genera in Aemodogryllini; the dorsal median lobe of the male genitalia has paired lateral sclerites at the basal area, the apical area does not divide; the dorsal lateral lobes of the male genitalia are broad and folded, much longer than dorsal median lobe, with the apical area slightly sclerotized, almost as wide as the basal area.

***Pseudotachycines volutus* Qin, Liu & Li, 2017**

*Pseudotachycines volutus* Qin, Liu & Li, 2017: 486.

(Figure 8)

**Material examined.** Holotype: ♂, China, Hunan, Yueyang, Forest Park of Fushoushan, 18~26.Ⅸ.2016, coll. Jiang et al., dep. SEMCAS. Paratypes: 1♂3♀, same data as for holotype; 2♂, China, Zhejiang, Tianmu Mountain, 31.Ⅷ.2012, coll. X.W. Liu et al., dep. SEMCAS. Other material: 1♂, China, Zhejiang, Linan, Tianmu Mountain, 15.Ⅷ.2018, coll. Y.J. Dou and Y.X. Zhen, dep. HBU; 1♀, China, Zhejiang, Linan, Tianmu Mountain, 12.Ⅷ.2018, coll. Y.J. Dou and Y.X. Zhen, dep. HBU.

**Distribution.** China (Hunan, Zhejiang).

**Figure 8:**
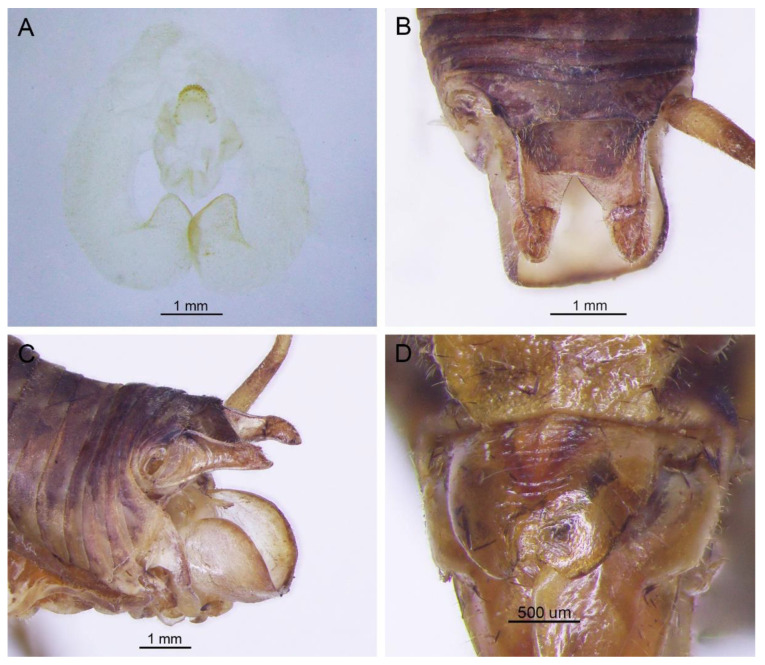
Type specimens of *Pseudotachycines volutus*. (**A**) dorsal view of male genitalia; (**B**,**C**) apex of abdomen: (**B**) dorsal view, (**C**) lateral view; (**D**) subgenital plate in ventral view.

**Remarks.** Because the type species of genus *Pseudotachycines* is moved to genus *Gymnaetoides*, *Pseudotachycines volutus* is redesignated as the type species of the genus *Pseudotachycines*. After examining the type specimen, we found that the line drawings of the male genitalia did not adequately represent the characters (Figures 14–16 in [14]). The photographs of the type specimens are shown in Figure 8.

**Figure 9:**
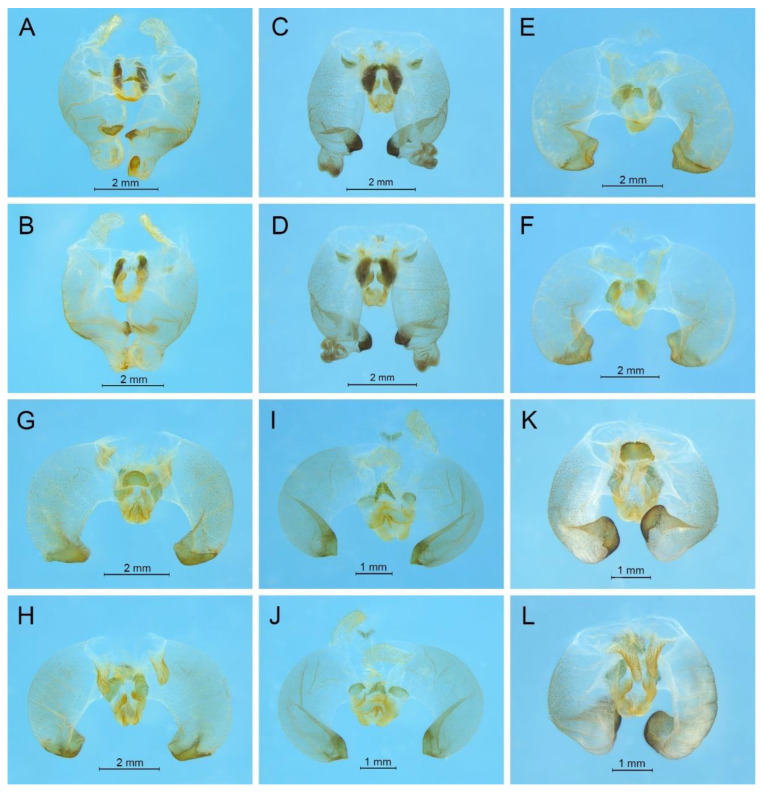
Male genitalia of *Pseudotachycines*. (**A**,**C**,**E**,**G**,**I**,**K**) dorsal view, (**B**,**D**,**F**,**H**,**J**,**L**) ventral view: (**A**,**B**) *Pseudotachycines procerus* Zhu & Shi sp. nov.; (**C**,**D**) *Pseudotachycines procerus guizhouensis* Zhu & Shi ssp. nov.; (**E**,**F**) *Pseudotachycines zhengi* Zhu & Shi sp. nov.; (**G**,**H**) *Pseudotachycines nephrus* Zhu & Shi sp. nov.; (**I**,**J**) *Pseudotachycines sagittus* Zhu & Shi sp. nov.; (**K**,**I**) *Pseudotachycines fengyangshanensis* Zhu & Shi sp. nov.

**Figure 10:**
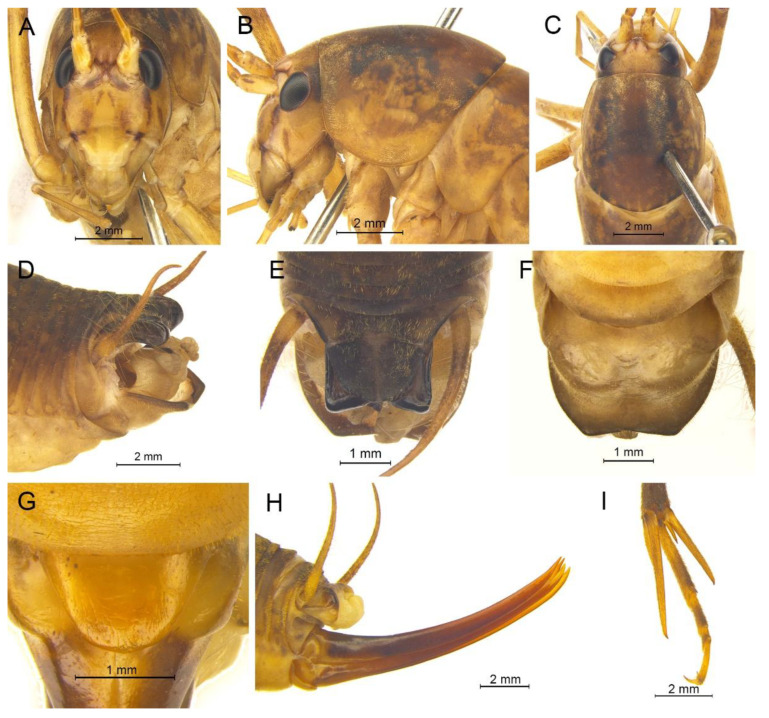
*Pseudotachycines procerus* Zhu & Shi sp. nov., male: (**A**–**C**) head and pronotum: (**A**) frontal view, (**B**) lateral view, (**C**) dorsal view; (**D**–**F**) apex of abdomen: (**D**) lateral view, (**E**) dorsal view, (**F**) ventral view; (**I**) hind tarsus in lateral view; female: (**G**) subgenital plate in ventral view; (**H**) ovipositor in lateral view.

***Pseudotachycines procerus* Zhu & Shi sp. nov.**

urn:lsid:zoobank.org:act:9BC06DD7-1F67-4F05-89F1-366023B49965

(Figure 9A,B and Figure 10)

**Material examined.** Holotype: ♂, China, Anhui, Yuexi, Yaoluoping, 18.Ⅸ.2019, coll. Y.Q. Li, dep. HBU. Paratypes: 3♂1♀, same data as for holotype.

**Etymology.** The name of the new species derives from the Latin word ‘*procer*’ (‘process’), referring to the subapical area of the dorsal lateral lobe of the male genitalia with a process on the dorsal surface.

**Diagnosis.** The new species can easily be distinguished from known congeneric species by the shape of the male genitalia and paraproct. The dorsal sclerite of the male genitalia is lunular and the lateral sclerite of the dorsal median lobe is oblong. The subapical area of the dorsal lateral lobe of the male genitalia has a process on the dorsal surface. The male paraproct is oblong in the lateral view.

**Description.** Male: Body medium sized in *Pseudotachycines*. Fastigium verticis with two conical tubercles, apices rounded, directing forward. Eyes ovoid, protruding forward; median ocellus oval, located between antennal sockets; lateral ocelli circular, situated on lateral margins of basal fastigium verticis. Apical segment of maxillary palpus much longer than subapical segment, apex inflated, globular.

Anterior margin of pronotum straight, posterior margin arcuate; lateral lobes longer than high, ventral margins arc shaped. Mesonotum and metanotum short, posterior margin of mesonotum arcuate, posterior margin of metanotum straight. Fore coxa with one small spine; femur unarmed on ventral surface, internal genicular lobe with one small spine, external genicular lobe with one long spine; tibia with two to three inner spines and two outer spines on ventral surface, apical area with one extero-dorsal spine and a pair of ventral spines, between the paired ventral spines with one small spine. Mid femur unarmed on ventral surface, internal and external genicular lobes each with one long spine; tibia with one inner spine and two outer spines on ventral surface, apical area with a pair of dorsal spines and a pair of ventral spines, between the paired ventral spines with one small spine. Hind femur with three to five inner spines on ventral surface; tibia with 57–60 inner spines and 55–56 outer spines on dorsal surface, with a pair of dorsal spines in subapical area, and at apex with a pair of dorsal spines and two pairs of ventral spines, intero-dorsal spine much longer than hind basitarsus. Hind basitarsus with one dorsal apical spine.

All abdominal tergites without process. Epiproct ligulate, paraproct extended backward, slightly longer than epiproct, oblong in lateral view. Cercus slender, conical, apex acute. Dorsal sclerite of genitalia small and lunular; basal area of dorsal median lobe with paired lateral sclerites, oblong, apical area not divided; dorsal lateral lobes broad and folded, much longer than dorsal median lobe, subapical area with a process on dorsal surface, apical area slightly sclerotized, almost as wide as basal area; ventral lateral lobes and ventral median lobe short. Subgenital plate quadrangular, basal half broad, apical half narrow, posterior margin slightly concave.

Female: Appearance is similar to male. Ovipositor longer than half the length of hind femur, curved upward, dorsal margin smooth, apical area of ventral margin denticulate. Subgenital plate quadrangular, basal area broad, gradually narrowing to apex, posterior margin blunt.

**Coloration.** Dorsal surface of body light brown, ventral surface yellowish brown. Face with four longitudinal black stripes.

**Measurements (mm).** Body: ♂15.3–17.2, ♀18.5; pronotum: ♂4.8–5.2, ♀6.4; fore femur: ♂6.8–7.0, ♀7.3; hind femur: ♂15.5–16.0, ♀17.7; hind tibia: ♂14.7–15.0, ♀16.8; hind basitarsus: ♂2.8–3.1, ♀2.8; ovipositor: 12.6.

**Distribution.** China (Anhui).

**Figure 11:**
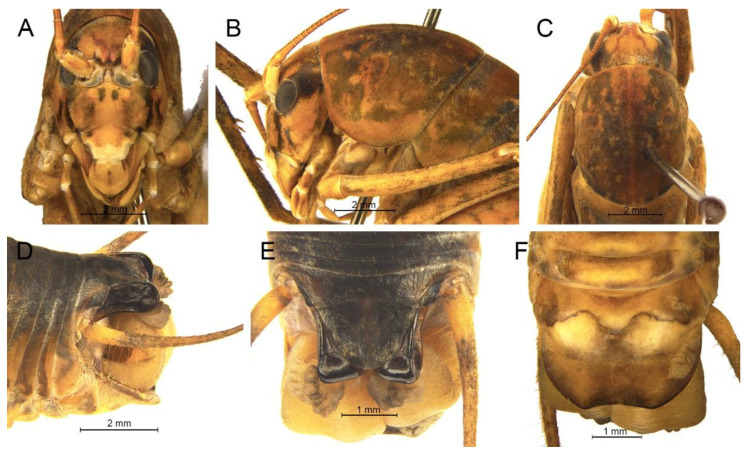
*Pseudotachycines procerus guizhouensis* Zhu & Shi ssp. nov., male: (**A**–**C**) head and pronotum: (**A**) frontal view, (**B**) lateral view, (**C**) dorsal view; (**D**–**F**) apex of abdomen: (**D**) lateral view, (**E**) dorsal view, (**F**) ventral view.

***Pseudotachycines procerus guizhouensis* Zhu & Shi ssp. nov.**

(Figure 9C,D and Figure 11)

**Material examined.** Holotype: ♂, China, Guizhou, Suiyang, Kuankuoshui, 13.Ⅷ.2017, coll. Q.D. Zhu and Y.Q. Li, dep. HBU. Paratype: 1♂, China, Guizhou, Daozhen, Dashahe, 7.Ⅷ.2017, coll. Q.D. Zhu and Y.Q. Li, dep. HBU.

**Etymology.** The name of the new species derives from the type locality.

**Diagnosis.** The species is very similar to *Pseudotachycines procerus* Zhu & Shi sp. nov., the slight differences are as follows: the lateral sclerite of the dorsal median lobe of the male genitalia is semicircular, and the hind femur has one to three inner spines on the ventral surface, which is less than three to five inner spines of the latter one.

**Description.** Male: Body medium sized in *Pseudotachycines*. Fastigium verticis with two conical tubercles, apices divided, directing forward. Eyes ovoid, protruding forward; median ocellus oval, located between antennal sockets; lateral ocelli circular, situated on lateral margins of basal fastigium verticis. Apical segment of maxillary palpus much longer than subapical segment, apex inflated, globular.

Pronotum long, anterior margin of disc straight, posterior margin arcuate; lateral lobes longer than high, ventral margins arc shaped. Mesonotum and metanotum short, posterior margin of mesonotum arcuate, posterior margin of metanotum straight. Fore coxa with one small spine; femur unarmed on ventral surface, internal genicular lobe with one small spine, external genicular lobe with one long spine; tibia with two inner spines and two outer spines on ventral surface, apical area with one extero-dorsal spine and a pair of ventral spines, between the paired ventral spines with one small spine. Mid femur unarmed on ventral surface, internal and external genicular lobes each with one long spine; tibia with one inner spine and two outer spines on ventral surface, apical area with a pair of dorsal spines and a pair of ventral spines, between the paired ventral spines with one small spine. Hind femur with one to three inner spines on ventral surface; tibia with 53–56 inner spines and 50–54 outer spines on dorsal surface, with a pair of dorsal spines in subapical area, and at apex with a pair of dorsal spines and two pairs of ventral spines, intero-dorsal spine much longer than hind basitarsus. Hind basitarsus with one dorsal apical spine.

All abdominal tergites without process. Epiproct ligulate, paraproct extended backward, slightly longer than epiproct, oblong in lateral view. Cercus slender, conical, apex acute. Dorsal sclerite of genitalia small and lunular, basal area slightly concave; basal area of dorsal median lobe with paired lateral sclerites, semicircular, apical area not divided; dorsal lateral lobes broad and folded, much longer than dorsal median lobe, subapical area with a process on dorsal surface, apical area sclerotized, almost as wide as basal area; ventral lateral lobes and ventral median lobe short. Subgenital plate quadrangular, basal half broad, apical half narrow, posterior margin slightly concave.

Female: Unknown.

**Coloration.** Dorsal surface of body light brown, ventral surface yellowish brown. Face with four longitudinal black stripes.

**Measurements (mm).** Body: ♂16.3–16.7; pronotum: ♂5.0–5.6; fore femur: ♂6.9–7.2; hind femur: ♂15.1–15.9; hind tibia: ♂15.1–15.5; hind basitarsus: ♂3.0–3.1.

**Distribution.** China (Guizhou).

**Remarks.** The species is very similar to *Pseudotachycines procerus* Zhu & Shi sp. nov. and there are only slight differences between them. However, all of the four species delimitation methods divide them into two MOTUs. Therefore, we regard it as a subspecies of *Pseudotachycines procerus* Zhu & Shi sp. nov.

**Figure 12:**
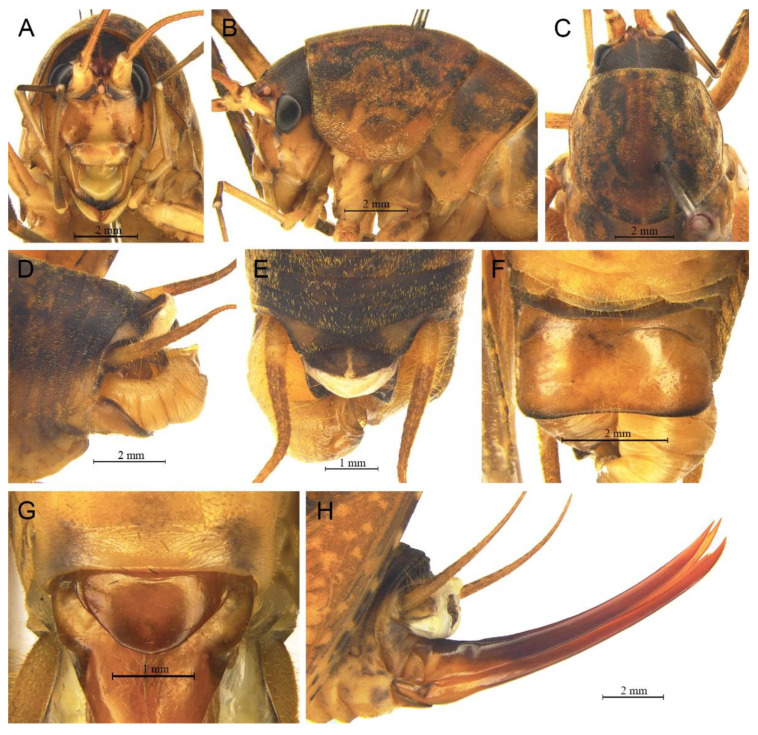
*Pseudotachycines zhengi* Zhu & Shi sp. nov., male: (**A**–**C**) head and pronotum: (**A**) frontal view, (**B**) lateral view, (**C**) dorsal view; (**D**–**F**) apex of abdomen: (**D**) lateral view, (**E**) dorsal view, (**F**) ventral view; female: (**G**) subgenital plate in ventral view; (**H**) ovipositor in lateral view.

***Pseudotachycines zhengi* Zhu & Shi sp. nov.**

urn:lsid:zoobank.org:act:E355BD1A-AE33-42BF-BE49-8BFFC44654CF

(Figure 9E,F and Figure 12)

**Material examined.** Holotype: ♂, China, Jiangxi, Lushan, Guling, 7.Ⅷ.2018, coll. T. Wang, dep. HBU. Paratypes: 3♂2♀, same data as for holotype. Other material: 5♂2♀, same data as for holotype; 1♂, China, Jiangxi, Lushan, Dayueshan, 3.Ⅷ.2018, coll. T. Wang, dep. HBU; 2♂, China, Zhejiang, Longquan, Fengyangshan, 18.Ⅸ.2019, coll. Y.X. Zhen, dep. HBU.

**Etymology.** The new species is named after Mr. Zhemin Zheng to commemorate his great contribution to the study of Chinese Orthoptera.

**Diagnosis.** The new species can easily be distinguished from known congeneric species by the shape of the male genitalia and paraproct. The dorsal sclerite of the male genitalia is nearly semicircular, with a concavity at the basal area and the apical area broadly concave. The lateral sclerite of the dorsal median lobe of the male genitalia is oblong. The male paraproct is semicircular in the lateral view.

**Description.** Male: Body medium sized in *Pseudotachycines*. Fastigium verticis with two conical tubercles, apices rounded, directing forward. Eyes ovoid, protruding forward; median ocellus oval, located between antennal sockets; lateral ocelli circular, situated on lateral margins of basal fastigium verticis. Apical segment of maxillary palpus much longer than subapical segment, apex inflated, globular.

Pronotum long, anterior margin straight, posterior margin arcuate; lateral lobes longer than high, ventral margins arc shaped. Mesonotum and metanotum short, posterior margin of mesonotum arcuate, posterior margin of metanotum straight. Fore coxa with one small spine; femur unarmed on ventral surface, internal genicular lobe with one small spine, external genicular lobe with one long spine; tibia with two inner spines and two outer spines on ventral surface, apical area with one extero-dorsal spine and a pair of ventral spines, between the paired ventral spines with one small spine. Mid femur unarmed on ventral surface, internal and external genicular lobes each with 1 long spine; tibia with one inner spine and two to three outer spines on ventral surface, apical area with a pair of dorsal spines and a pair of ventral spines, between the paired ventral spines with one small spine. Hind femur with two inner spines on ventral surface; tibia with 56–59 inner spines and 59–62 outer spines on dorsal surface, with a pair of dorsal spines in subapical area, and at apex with a pair of dorsal spines and two pairs of ventral spines, intero-dorsal spine much longer than hind basitarsus. Hind basitarsus with one dorsal apical spine.

All abdominal tergites without process. Epiproct lingulate; paraproct slightly longer than epiproct, semicircular in lateral view. Cercus slender, conical, apex acute. Dorsal sclerite of genitalia nearly semicircular, basal area with a concavity, apical area broadly concave; basal area of dorsal median lobe with paired lateral sclerites, oblong, apical area not divided; dorsal lateral lobes broad and folded, much longer than dorsal median lobe, apical area weakly sclerotized, almost as wide as basal area; ventral median lobe and ventral lateral lobes short. Subgenital plate quadrangular, wide, posterior margin straight.

Female: Appearance is similar to male. Ovipositor longer than half the length of hind femur, dorsal margin smooth, apical area of ventral margin denticulate. Subgenital plate semicircular, posterior margin blunt.

**Coloration.** Dorsal surface of body brown, ventral surface yellowish brown. Face with four longitudinal black stripes.

**Measurements (mm).** Body: ♂15.2–18.0, ♀17.8–20.1; pronotum: ♂5.8–7.1, ♀6.0–7.0; fore femur: ♂7.3–8.8, ♀7.0–8.3; hind femur: ♂17.0–19.0, ♀18.1–19.0; hind tibia: ♂15.0–16.9, ♀17.8–19.0; hind basitarsus: ♂4.0–4.8, ♀3.2–4.0; ovipositor: 12.9–15.1.

**Distribution.** China (Jiangxi, Zhejiang).

**Figure 13:**
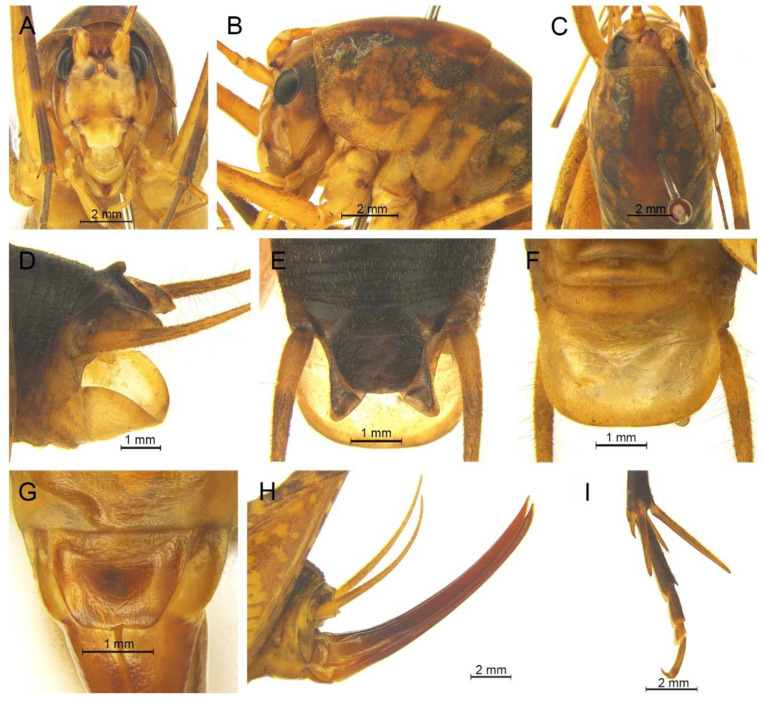
*Pseudotachycines nephrus* Zhu & Shi sp. nov., male: (**A**–**C**) head and pronotum: (**A**) frontal view, (**B**) lateral view, (**C**) dorsal view; (**D**–**F**) apex of abdomen: (**D**) lateral view, (**E**) dorsal view, (**F**) ventral view; (**I**) hind tarsus in lateral view; female: (**G**) subgenital plate in ventral view; (**H**) ovipositor in lateral view.

***Pseudotachycines nephrus* Zhu & Shi sp. nov.**

urn:lsid:zoobank.org:act:31599921-80F7-4902-9FEC-0FCDCA3B848F

(Figure 9G,H and Figure 13)

**Material examined.** Holotype: ♂, China, Zhejiang, Linan, Qingliangfeng, 4.Ⅹ.2019, coll. Q.D. Zhu, dep. HBU. Paratypes: 2♀, same data as for holotype.

**Etymology.** The name of the new species is the Greek word ‘*nephr*’ (nephroid), referring to the nephroid lateral sclerites of the dorsal median lobe of the male genitalia.

**Diagnosis.** The new species is similar to *Pseudotachycines zhengi* Zhu & Shi sp. nov., but it can easily be distinguished from the latter one by the shape of the male genitalia and female subgenital plate. The dorsal sclerite of the male genitalia is semicircular and the lateral sclerite of the dorsal median lobe is nephroid. The female subgenital plate is trapezoid, with slightly concave posterior margin.

**Description.** Male: Body medium sized in *Pseudotachycines*. Fastigium verticis with two conical tubercles, apices rounded, directing forward. Eyes ovoid, protruding forward; median ocellus oval, located between antennal sockets; lateral ocelli circular, situated on lateral margins of basal fastigium verticis. Apical segment of maxillary palpus much longer than subapical segment, apex truncated.

Pronotum long, anterior margin straight, posterior margin arcuate; lateral lobes longer than high, ventral margins arc shaped. Mesonotum and metanotum short, posterior margin of mesonotum arcuate, posterior margin of metanotum straight. Fore coxa with one small spine; femur unarmed on ventral surface, internal genicular lobe with one small spine, external genicular lobe with one long spine; tibia with two inner spines and two outer spines on ventral surface, apical area with one extero-dorsal spine and a pair of ventral spines, between the paired ventral spines with one small spine. Mid femur unarmed on ventral surface, internal and external genicular lobes each with one long spine; tibia with one inner spine and two outer spines on ventral surface, apical area with a pair of dorsal spines and a pair of ventral spines, between the paired ventral spines with one small spine. Hind femur with three to five inner spines on ventral surface, internal genicular lobe with one small spine, external genicular lobe unarmed; tibia with 55–57 inner spines and 55–57 outer spines on dorsal surface, with a pair of dorsal spines in subapical area, and at apex with a pair of dorsal spines and two pairs of ventral spines, intero-dorsal spine much longer than hind basitarsus. Hind basitarsus with one dorsal apical spine.

Posterior margins of all abdominal tergites straight. Epiproct semicircular; paraproct much longer than epiproct, triangular in lateral view. Cercus slender, conical, apex acute. Dorsal sclerite of genitalia semicircular; basal area of dorsal median lobe with paired lateral sclerites, nephroid, apical area not divided; dorsal lateral lobes broad and folded, much longer than dorsal median lobe, apical area weakly sclerotized, almost as wide as basal area; ventral median lobe and ventral lateral lobes short. Subgenital plate quadrangular, posterior margin straight.

Female: Appearance is similar to male. Ovipositor longer than half the length of hind femur, curved upward, dorsal margin smooth, apical area of ventral margin denticulate. Subgenital plate trapezoid, posterior margin slightly concave.

**Coloration.** Dorsal surface of body light brown, with yellow spots, ventral surface yellowish brown. Eyes black.

**Measurements (mm).** Body: ♂14.1, ♀17.4–18.3; pronotum: ♂6.6, ♀7.3–8.0; fore femur: ♂8.4, ♀9.0–11.2; hind femur: ♂19.5, ♀21.6–23.2; hind tibia: ♂18.8, ♀21.4–22.5; hind basitarsus: ♂3.3, ♀4.1–4.6; ovipositor: 13.0–15.9.

**Distribution.** China (Zhejiang).

**Figure 14:**
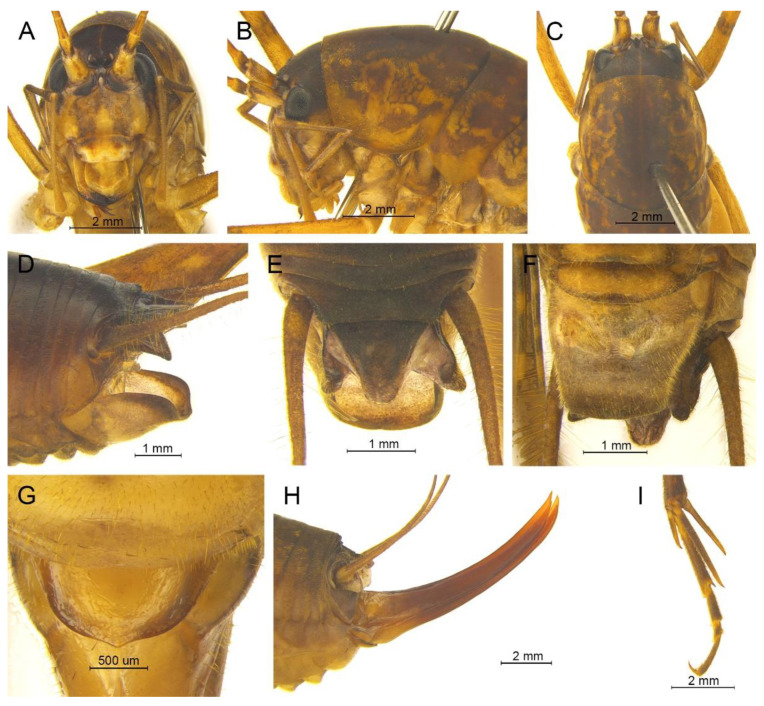
*Pseudotachycines sagittus* Zhu & Shi sp. nov., male: (**A**–**C**) head and pronotum: (**A**) frontal view, (**B**) lateral view, (**C**) dorsal view; (**D**–**F**) apex of abdomen: (**D**) lateral view, (**E**) dorsal view, (**F**) ventral view; (**I**) hind tarsus in lateral view; female: (**G**) subgenital plate in ventral view; (**H**) ovipositor in lateral view.

***Pseudotachycines sagittus* Zhu & Shi sp. nov.**

urn:lsid:zoobank.org:act:65264F0A-DAC2-4CD0-BE06-0097493456ED

(Figure 9I,J and Figure 14)

**Material examined.** Holotype: ♂, China, Yunnan, Yiliang, Chaotianma, 11.Ⅷ.2020, coll. T. Wang, dep. HBU. Paratypes: 2♀, same data as for holotype.

**Etymology.** The name of the new species derives from the Latin word ‘*sagitt*’, referring to the sagittate-shaped dorsal sclerite of the male genitalia.

**Diagnosis.** The new species can easily be distinguished from known congeneric species by the shape of the male genitalia and epiproct. The dorsal sclerite of the male genitalia is sagittate and the lateral sclerite of the dorsal median lobe of the male genitalia is oval. The male epiproct is nearly triangular.

**Description.** Male: Body medium sized in *Pseudotachycines*. Fastigium verticis short, with two conical tubercles, apices rounded, directing forward. Eyes ovoid, protruding forward; median ocellus oval, located between antennal sockets; lateral ocelli circular, situated on lateral margins of basal fastigium verticis. Apical segment of maxillary palpus much longer than subapical segment, apex truncated.

Pronotum long, anterior margin straight, posterior margin arcuate; lateral lobes longer than high, ventral margins arc shaped. Mesonotum and metanotum short, posterior margin of mesonotum arcuate, posterior margin of metanotum straight. Fore coxa with one small spine; femur unarmed on ventral surface, internal genicular lobe unarmed, external genicular lobe with one long spine; tibia with two inner spines and two outer spines on ventral surface, apical area with one extero-dorsal spine and a pair of ventral spines, between the paired ventral spines with one small spine. Mid femur unarmed on ventral surface, internal and external genicular lobes each with one long spine; tibia with one inner spine and two outer spines on ventral surface, apical area with a pair of dorsal spines and a pair of ventral spines, between the paired ventral spines with one small spine. Hind femur unarmed on ventral surface, internal genicular lobe with one small spine, external genicular lobe unarmed; tibia with 47–52 inner spines and 49–50 outer spines on dorsal surface, with a pair of dorsal spines in subapical area, and at apex with a pair of dorsal spines and two pairs of ventral spines, intero-dorsal spine nearly equal in length to hind basitarsus. Hind basitarsus with one dorsal apical spine.

All abdominal tergites without process. Epiproct nearly triangular, apex blunt; paraproct triangular in lateral view. Cercus slender, conical, apex acute. Dorsal sclerite of genitalia sagittate; basal area of dorsal median lobe with paired lateral sclerites, oval, apical area not divided; dorsal lateral lobes broad and folded, much longer than dorsal median lobe, apical area weakly sclerotized, almost as wide as basal area; ventral median lobe and ventral lateral lobes short. Subgenital plate quadrangular, posterior margin arcuate.

Female: Appearance is similar to male. Ovipositor longer than half the length of hind femur, curved upward, dorsal margin smooth, apical area of ventral margin denticulate. Subgenital plate semicircular, posterior margin slightly prominent.

**Coloration.** Body light brown, face with four longitudinal brown stripes, eyes black.

**Measurements (mm).** Body: ♂13.7, ♀15.6–15.8; pronotum: ♂4.6, ♀5.0–5.7; fore femur: ♂7.2, ♀7.0–7.5; hind femur: ♂15.2, ♀15.6–17.4; hind tibia: ♂15.3, ♀15.5–16.9; hind basitarsus: ♂3.0, ♀2.6–2.7; ovipositor: 9.7–9.8.

**Distribution.** China (Yunnan).

**Figure 15:**
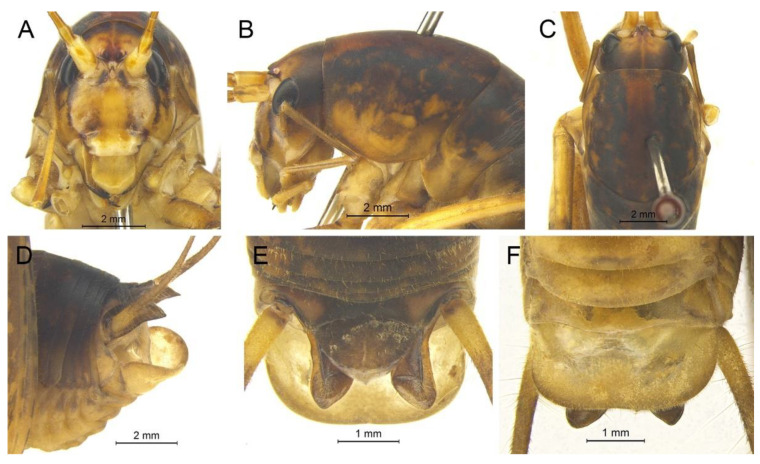
*Pseudotachycines fengyangshanensis* Zhu & Shi sp. nov., male: (**A**–**C**) head and pronotum: (**A**) frontal view, (**B**) lateral view, (**C**) dorsal view; (**D**–**F**) apex of abdomen: (**D**) lateral view, (**E**) dorsal view, (**F**) ventral view.

***Pseudotachycines fengyangshanensis* Zhu & Shi sp. nov.**

urn:lsid:zoobank.org:act:1AD154B4-D04F-47F7-988D-8C568B94F515

(Figure 9K,L and Figure 15)

**Material examined.** Holotype: ♂, China, Zhejiang, Longquan, Fengyangshan, 18.Ⅸ.2019, coll. Y.X. Zhen, dep. HBU. Paratype: 1♂, same data as for holotype.

**Etymology.** The name of the new species derives from the type locality.

**Diagnosis.** The new species is most similar to *Pseudotachycines nephrus* Zhu & Shi sp. nov., but it can be distinguished by the dorsal sclerite of the male genitalia semicircular, with the basal area slightly concave and the apex truncate, and the lateral sclerite of the dorsal median lobe of the male genitalia oblong.

**Description.** Male: Body medium sized in *Pseudotachycines*. Fastigium verticis with two conical tubercles, apices divided, directing forward. Eyes ovoid, protruding forward; median ocellus oval, located between antennal sockets; lateral ocelli circular, situated on lateral margins of basal fastigium verticis. Apical segment of maxillary palpus much longer than subapical segment, apex inflated, globular.

Pronotum long, anterior margin of disc straight, posterior margin arcuate; lateral lobes longer than high, ventral margins arc shaped. Mesonotum and metanotum short, posterior margin of mesonotum arcuate, posterior margin of metanotum straight. Fore coxa with one small spine; femur unarmed on ventral surface, internal genicular lobe with one small spine, external genicular lobe with one long spine; tibia with two inner spines and two outer spines on ventral surface, apical area with one extero-dorsal spine and a pair of ventral spines, between the paired ventral spines with one small spine. Mid femur unarmed on ventral surface, internal and external genicular lobes each with one long spine; tibia with one inner spine and two outer spines on ventral surface, apical area with a pair of dorsal spines and a pair of ventral spines, between the paired ventral spines with one small spine. Hind femur with zero to four spines, internal genicular lobe with one small spine; tibia with 51 inner spines and 58 outer spines on dorsal surface, with a pair of dorsal spines in subapical area, and at apex with a pair of dorsal spines and two pairs of ventral spines, intero-dorsal spine much longer than hind basitarsus. Hind basitarsus with one dorsal apical spine.

All abdominal tergites without process. Epiproct nearly semicircular; paraproct much longer than epiproct, oblong in lateral view. Cercus slender, conical, apex acute. Dorsal sclerite of genitalia semicircular, basal area slightly concave, apex truncate; basal area of dorsal median lobe with paired lateral sclerites, oblong, apical area not divided; dorsal lateral lobes broad and folded, much longer than dorsal median lobe, apical area weakly sclerotized, almost as wide as basal area; ventral median lobe and ventral lateral lobes short. Subgenital plate quadrangular, transverse and wide, posterior margin straight.

Female: Unknown.

**Coloration.** Body brown on dorsal surface and yellowish brown on ventral surface. Face with four longitudinal black stripes. Basal half of the hind femur with pinnate black stripes.

**Measurements (mm).** Body: ♂15.3; pronotum: ♂6.1; fore femur: ♂7.8; hind femur: ♂17.1; hind tibia: ♂16.9; hind basitarsus: ♂3.3.

**Distribution.** China (Zhejiang).

**Genus *Homotachycines* Zhu & Shi gen. nov.**

urn:lsid:zoobank.org:act:D01BD682-64B3-4466-A591-DA784748B724

**Type species.***Homotachycines quadratus* Zhu & Shi sp. nov., here designated.

**Etymology.** The name of the new genus refers to the Latin word ‘*homo*’ and the genus *Tachycines*.

**Diagnosis.** Body is medium sized in Aemodogryllini. The male epiproct and paraproct are normally developed. The male genitalia have unpaired dorsal sclerite; the dorsal median lobe of the male genitalia has paired lateral sclerites at the basal area, the apical area does not divide; the dorsal lateral lobes of the male genitalia are little longer than the dorsal median lobe, the basal area broad, gradually narrowing to apices, the apical area sclerotized.

***Homotachycines acutilobatus* (Gorochov, 2010) comb. nov.**

*Diestrammena* (*Gymnaeta*) *acutilobata* Gorochov, 2010a: 10; Gorochov, 2010b: 13.

**Figure 16:**
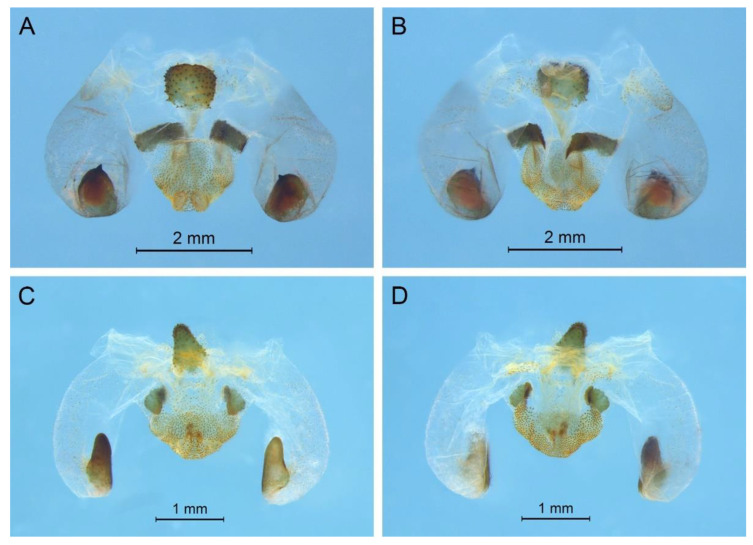
Male genitalia of *Homotachycines*. (**A**,**C**) dorsal view, (**B**,**D**) ventral view: (**A**,**B**) *Homotachycines acutilobatus* comb. nov.; (**C**,**D**) *Homotachycines ovalilobatus* comb. nov.

**Figure 17:**
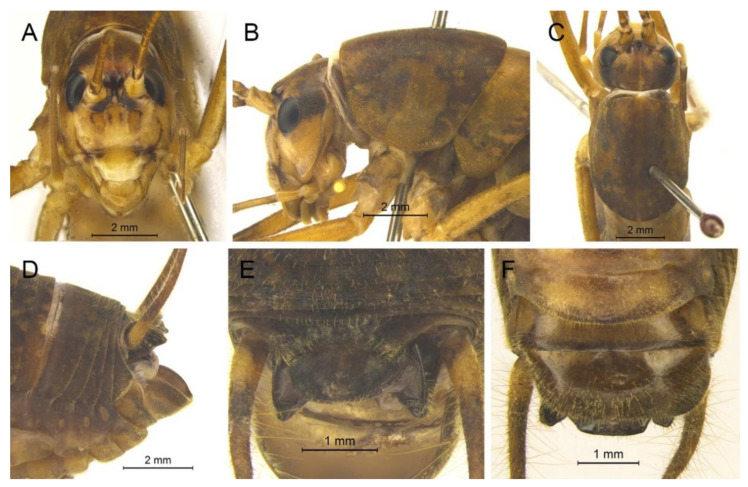
*Homotachycines acutilobatus* comb. nov., male: (**A**–**C**) head and pronotum: (**A**) frontal view, (**B**) lateral view, (**C**) dorsal view; (**D**–**F**) apex of abdomen: (**D**) lateral view, (**E**) dorsal view, (**F**) ventral view.

*Gymnaetoides acutilobatus*: Qin, Liu & Li, 2017: 189.

(Figure 16A,B and Figure 17)

**Material examined.** 1♂, China, Chongqing, Wushan, Guanyang, 22.Ⅶ.2021, coll. L.Y. Wang, dep. HBU; 4♂, China, Hubei, Shennongjia, Huangbaiqian, 17.Ⅷ.2019, coll. P. Wang, dep. HBU.

**Distribution.** China (Chongqing, Hubei).

**Remarks.** The species was transferred to genus *Gymnaetoides* according to the sclerotized dorsal lateral lobes of the male genitalia [13]. However, some characters of the species are inconsistent with *Gymnaetoides*: the apical area of the dorsal median lobe of the male genitalia does not divide, and the dorsal lateral lobe of the male genitalia is a little longer than the dorsal median lobe. Therefore, we transfer it into the genus *Homotachycines* based on morphological and molecular evidence.

***Homotachycines ovalilobatus* (Gorochov, 2010) comb. nov.**

*Diestrammena* (*Gymnaeta*) *ovalilobata* Gorochov, 2010a: 11.

**Figure 18:**
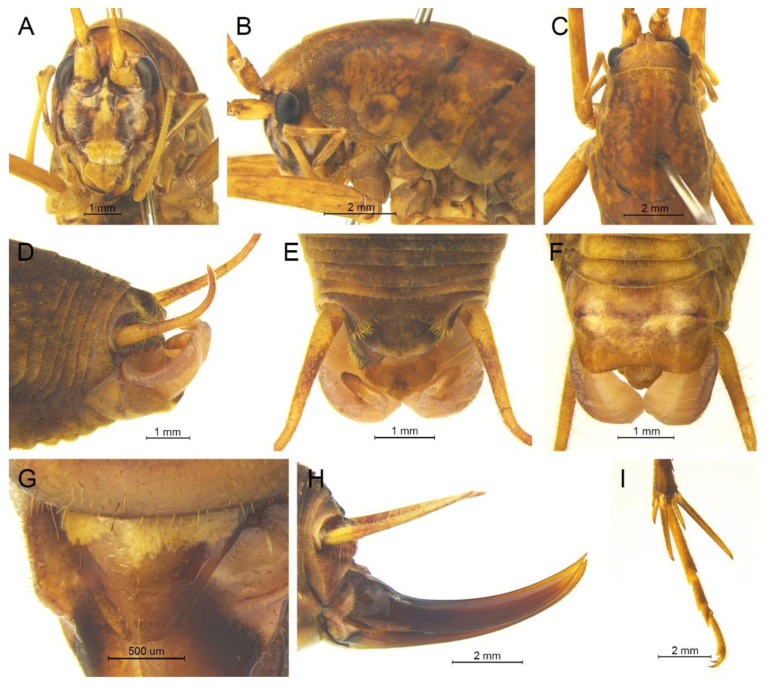
*Homotachycines ovalilobatus* comb. nov., male: (**A**–**C**) head and pronotum: (**A**) frontal view, (**B**) lateral view, (**C**) dorsal view; (**D**–**F**) apex of abdomen: (**D**) lateral view, (**E**) dorsal view, (**F**) ventral view; (**I**) hind tarsus in lateral view; female: (**G**) subgenital plate in ventral view; (**H**) ovipositor in lateral view.

*Pseudotachycines ovalilobatus*: Qin, Liu & Li, 2017: 488.

(Figure 16C,D and Figure 18)

**Material examined.** 2♂2♀, China, Hubei, Baokang, Houping, 8.Ⅷ.2020, coll. G.L. Xie, dep. HBU; 2♂2♀, China, Hubei, Shennongjia, Huangbaiqian, 17.Ⅷ.2019, coll. P. Wang, dep. HBU.

**Distribution.** China (Hubei).

**Remarks.** The species was moved to the genus *Pseudotachycines* according to the generic diagnosis [14]. However, there are some characters that differ from *Pseudotachycines*: the male paraproct is normally developed; the dorsal lateral lobes of the male genitalia are little longer than dorsal median lobe, with the basal area broad, gradually narrowing to apices, the apical area sclerotized, and oval lobate. Therefore, it should be transferred into *Homotachycines* based on morphological and molecular evidence.

***Homotachycines trapezialis* (Qin, Liu & Li, 2017) comb. nov.**

*Pseudotachycines trapezialis* Qin, Liu & Li, 2017: 487.

(Figure 19)

**Material examined.** Holotype: ♂, China, Henan, Funiu Mountain, 2.Ⅷ.2009, coll. W.B. Zhu, dep. SEMCAS. Paratype: 1♀, same data as for holotype.

**Distribution.** China (Henan).

**Figure 19:**
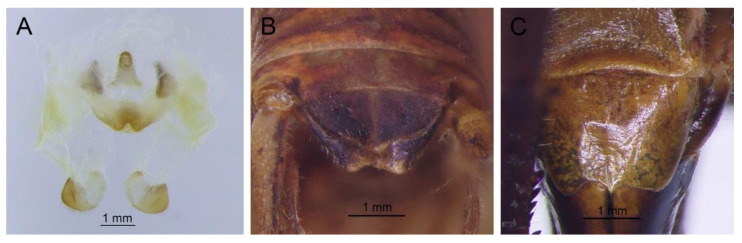
*Homotachycines trapezialis* comb. nov. (type specimens), male: (**A**) genitalia in dorsal view; (**B**) apex of abdomen in dorsal view; female: (**C**) subgenital plate in ventral view.

**Remarks.** The line drawing of the male genitalia of this species did not adequately represent the characters (Figure 19 in [14]). The photographs of the type specimens are shown in Figure 19. The male paraproct is normally developed. The apical area of the dorsal median lobe of the male genitalia does not divide. The dorsal lateral lobes of the male genitalia are longer than the dorsal median lobe, the basal area is broad, gradually narrowing to apices, and the apical area is sclerotized. According to these characters, this species should be moved to genus *Homotachycines*. The species is similar to *Homotachycines ovalilobatus* comb. nov. in the shape of the male genitalia, but the female subgenital plate is quite different.

**Figure 20:**
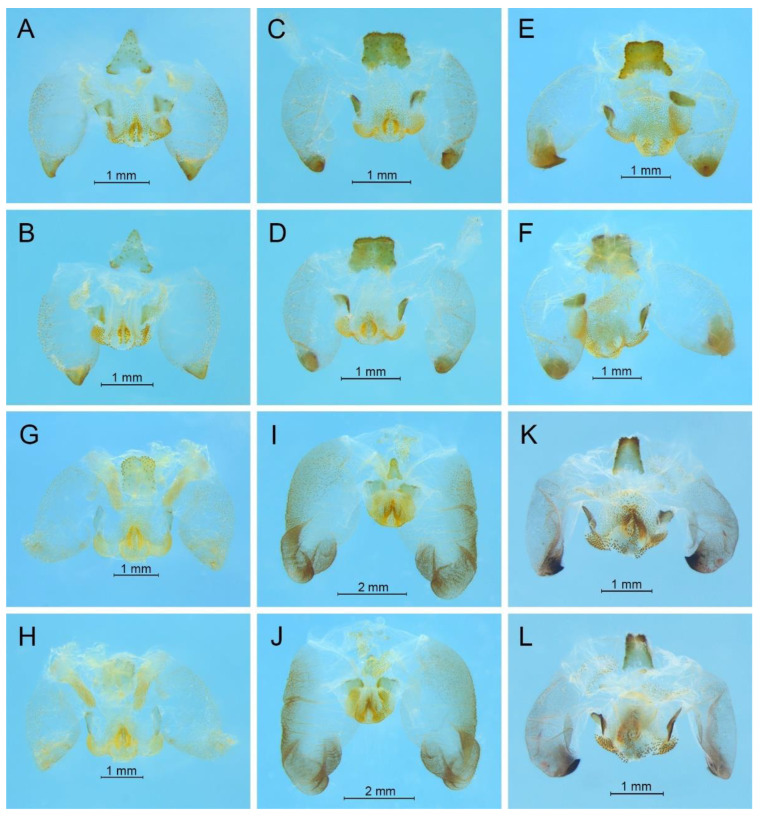
Male genitalia of *Homotachycines*. (**A**,**C**,**E**,**G**,**I**,**K**) dorsal view, (**B**,**D**,**F**,**H**,**J**,**L**) ventral view: (**A**,**B**) *Homotachycines triangulus* Zhu & Shi sp. nov.; (**C**,**D**) *Homotachycines quadratus* Zhu & Shi sp. nov.; (**E**,**F**) *Homotachycines baokangensis* Zhu & Shi sp. nov.; (**G**,**H**) *Homotachycines fusus* Zhu & Shi sp. nov.; (**I**,**J**) *Homotachycines concavus* Zhu & Shi sp. nov.; (**K**,**L**) *Homotachycines qinlingensis* Zhu & Shi sp. nov.

**Figure 21:**
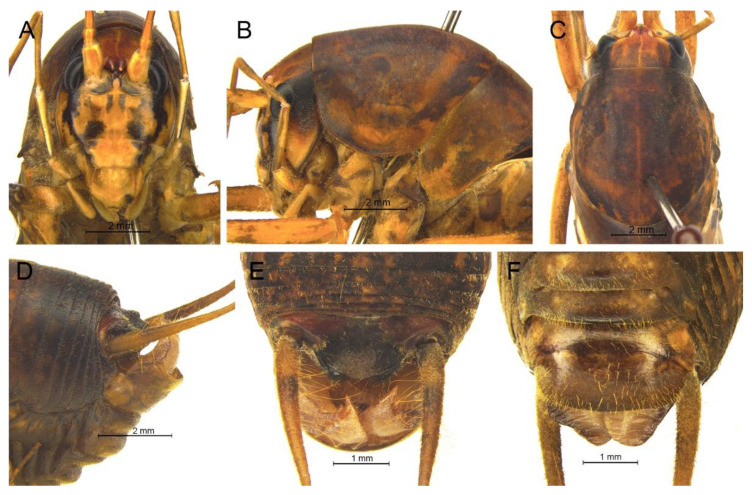
*Homotachycines triangulus* Zhu & Shi sp. nov., male: (**A**–**C**) head and pronotum: (**A**) frontal view, (**B**) lateral view, (**C**) dorsal view; (**D**–**F**) apex of abdomen: (**D**) lateral view, (**E**) dorsal view, (**F**) ventral view.

***Homotachycines triangulus* Zhu & Shi sp. nov.**

urn:lsid:zoobank.org:act:85851298-45FA-4F51-9858-85EC8A4E7F3A

(Figure 20A,B and Figure 21)

**Material examined.** Holotype: ♂, China, Hubei, Shennongjia, Maohu Protection Station, 15.Ⅷ.2018, coll. P. Wang, dep. HBU. Paratype: 1♂, China, Hubei, Shennongjia, Chaoshuihe, 8.Ⅷ.2018, coll. P. Wang, dep. HBU.

**Etymology.** The name of the new species derives from the Latin word ‘*tri*’ (triple) and ‘*angul*’ (angle), referring to the triangular dorsal sclerite of the male genitalia.

**Diagnosis.** The new species differs from other congeneric species by the shapes of the dorsal sclerite of the male genitalia and the lateral sclerite of the dorsal median lobe of the male genitalia. The dorsal sclerite of the male genitalia is triangular, with a broadly concave apical area, and the lateral sclerite of the dorsal median lobe is quadrangular.

**Description.** Male: Body medium sized in *Homotachycines*. Fastigium verticis with two conical tubercles, apices rounded, directing forward. Eyes ovoid, protruding forward; median ocellus oval, located between antennal sockets; lateral ocelli circular, situated on lateral margins of basal fastigium verticis. Apical segment of maxillary palpus much longer than subapical segment, apex truncated.

Pronotum long, anterior margin straight, posterior margin arcuate; lateral lobes longer than high, ventral margins arc shaped. Mesonotum and metanotum short, posterior margin of mesonotum arcuate, posterior margin of metanotum straight. Fore coxa with one small spine; femur unarmed on ventral surface, internal genicular lobe with one small spine, external genicular lobe with one long spine; tibia with two inner spines and two outer spines on ventral surface, apical area with one extero-dorsal spine and a pair of ventral spines, between the paired ventral spines with one small spine. Mid femur unarmed on ventral surface, internal and external genicular lobes each with one long spine; tibia with one inner spine and two outer spines on ventral surface, apical area with a pair of dorsal spines and a pair of ventral spines, between the paired ventral spines with one small spine. Hind femur with zero to one inner spine on ventral surface; tibia with 52–60 inner spines and 53–57 outer spines on dorsal surface, with a pair of dorsal spines in subapical area, and at apex with a pair of dorsal spines and two pairs of ventral spines, intero-dorsal spine distinctly longer than hind basitarsus. Hind basitarsus with one dorsal apical spine.

Posterior margins of all abdominal tergites straight. Epiproct semicircular; paraproct simple, triangular in lateral view. Cercus slender, conical, apex acute. Dorsal sclerite of genitalia triangular, apical area broadly concave; basal area of dorsal median lobe with paired lateral sclerites, quadrangular, apical area not divided; dorsal lateral lobes little longer than dorsal median lobe, basal area broad, gradually narrowing to apices, apical area sclerotized, hooked; ventral median lobe and ventral lateral lobes very short. Subgenital plate quadrangular, wide, posterior margin straight.

Female: Unknown.

**Coloration.** Body light brown. Eyes black, ocelli yellow. Face with four longitudinal black stripes, apical areas of all femora black.

**Measurements (mm).** Body: ♂15.5–17.8; pronotum: ♂5.8–6.0; fore femur: ♂6.2–7.0; hind femur: ♂15.3–16.2; hind tibia: ♂14.5–16.0; hind basitarsus: ♂3.0–3.2.

**Distribution.** China (Hubei).

**Figure 22:**
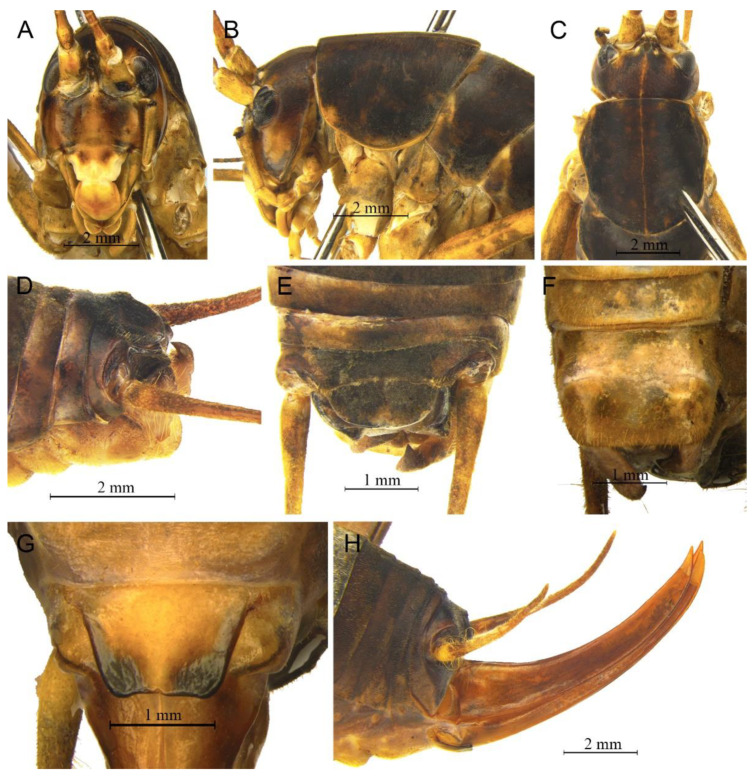
*Homotachycines quadratus* Zhu & Shi sp. nov., male: (**A**–**C**) head and pronotum: (**A**) frontal view, (**B**) lateral view, (**C**) dorsal view; (**D**–**F**) apex of abdomen: (**D**) lateral view, (**E**) dorsal view, (**F**) ventral view; female: (**G**) subgenital plate in ventral view; (**H**) ovipositor in lateral view.

***Homotachycines quadratus* Zhu & Shi sp. nov.**

urn:lsid:zoobank.org:act:2E728F0F-915C-4C36-98E3-B1CC9E531A7D

(Figure 20C,D and Figure 22)

**Material examined.** Holotype: ♂, China, Hubei, Shennongjia, Banbiyan, 14.Ⅷ.2018, coll. P. Wang, dep. HBU. Paratypes: 2♀, same data as for holotype. Other material: 1♀, same data as for holotype.

**Etymology.** The name of the new species is the Latin word ‘*quadrat*’ (quadrate), referring to the dorsal sclerite of the male genitalia having a quadrate shape.

**Diagnosis.** The new species is similar to *Homotachycines acutilobatus* comb. nov., but it can be distinguished from the latter one by the apical area of the dorsal sclerite of the male genitalia slightly concave and the lateral sclerite of the dorsal median lobe of the male genitalia crescent shaped.

**Description.** Male: Body medium sized in *Homotachycines*. Fastigium verticis short, with two conical tubercles, directing forward. Eyes ovoid, protruding forward; median ocellus oval, located between antennal sockets; lateral ocelli circular, situated on lateral margins of basal fastigium verticis. Apical segment of maxillary palpus much longer than subapical segment, apex truncated.

Pronotum long, anterior margin straight, posterior margin arcuate; lateral lobes longer than high, ventral margins arc shaped. Mesonotum and metanotum short, posterior margin of mesonotum arcuate, posterior margin of metanotum straight. Fore coxa with one small spine; femur unarmed on ventral surface, internal genicular lobe unarmed, external genicular lobe with 1 long spine; tibia with two inner spines and two outer spines on ventral surface, apical area with one extero-dorsal spine and a pair of ventral spines, between the paired ventral spines with one small spine. Mid femur unarmed on ventral surface, internal and external genicular lobes each with one long spine; tibia with one inner spine and one outer spine on ventral surface, apical area with a pair of dorsal spines and a pair of ventral spines, between the paired ventral spines with one small spine. Hind femur unarmed on ventral surface; tibia with 68–75 inner spines and 70–74 outer spines on dorsal surface, with a pair of dorsal spines in subapical area, and at apex with a pair of dorsal spines and two pairs of ventral spines, intero-dorsal spine slightly shorter than hind basitarsus. Hind basitarsus with one dorsal apical spine.

Posterior margins of all abdominal tergites straight. Epiproct semicircular; paraproct simple, triangular in lateral view. Cercus slender, conical, apex acute. Dorsal sclerite of genitalia quadrate, apical area slightly concave; basal area of dorsal median lobe with paired lateral sclerites, crescent-shaped, apical area not divided; dorsal lateral lobes little longer than dorsal median lobe, basal area broad, gradually narrowing to apices, apical area sclerotized, hooked; ventral median lobe and ventral lateral lobes short. Subgenital plate trapezoidal, posterior margin straight.

Female: Appearance is similar to male. Ovipositor short, curved upward, dorsal margin smooth, apical area of ventral margin denticulate. Subgenital plate trapezoid, posterior margin straight.

**Coloration.** Dorsal surface of body brown, ventral surface yellowish brown. Legs with black stripes.

**Measurements (mm).** Body: ♂17.0, ♀17.0–17.5; pronotum: ♂4.8, ♀5.0–6.0; fore femur: ♂8.0, ♀8.8–9.0; hind femur: ♂15.6, ♀16.0–18.2; hind tibia: ♂16.2, ♀16.2–18.0; hind basitarsus: ♂3.2, ♀3.5–4.0; ovipositor: 9.0–10.4.

**Distribution.** China (Hubei).

**Figure 23:**
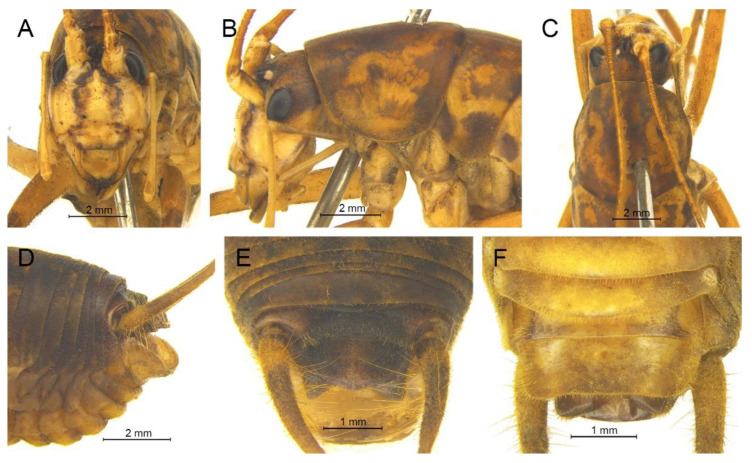
*Homotachycines baokangensis* Zhu & Shi sp. nov., male: (**A**–**C**) head and pronotum: (**A**) frontal view, (**B**) lateral view, (**C**) dorsal view; (**D**–**F**) apex of abdomen: (**D**) lateral view, (**E**) dorsal view, (**F**) ventral view.

***Homotachycines baokangensis* Zhu & Shi sp. nov.**

urn:lsid:zoobank.org:act:CE6C4893-12E1-4C96-A2DD-5D67369ED27B

(Figure 20E,F and Figure 23)

**Material examined.** Holotype: ♂, China, Hubei, Baokang, Houping, 8.Ⅷ.2020, coll. G.L. Xie, dep. HBU.

**Etymology.** The name of the new species refers to the type locality.

**Diagnosis.** The new species can be distinguished from known congeneric species by the shape of the male genitalia. The dorsal sclerite of the male genitalia is quadrangular, with the basal half rectangular and the apical half wide and slightly concave. The lateral sclerite of the dorsal median lobe of the male genitalia is oblong.

**Description.** Male: Body medium sized in *Homotachycines*. Fastigium verticis with two conical tubercles, apices rounded, directing forward. Eyes ovoid, protruding forward; median ocellus oval, located between antennal sockets; lateral ocelli circular, situated on lateral margins of basal fastigium verticis. Apical segment of maxillary palpus much longer than subapical segment, apex inflated, globular.

Pronotum long, anterior margin straight, posterior margin arcuate; lateral lobes longer than high, ventral margins arc shaped. Mesonotum and metanotum short, posterior margin of mesonotum arcuate, posterior margin of metanotum straight. Fore coxa with one small spine; femur unarmed on ventral surface, internal genicular lobe with 1 small spine, external genicular lobe with one long spine; tibia with two inner spines and two outer spines on ventral surface, apical area with one extero-dorsal spine and a pair of ventral spines, between the paired ventral spines with one small spine. Mid femur unarmed on ventral surface, internal and external genicular lobes each with one long spine; tibia with one inner spine and two outer spines on ventral surface, apical area with a pair of dorsal spines and a pair of ventral spines, between the paired ventral spines with one small spine. Hind femur with two inner spines on ventral surface, internal genicular lobe unarmed; tibia with 79 inner spines and 74 outer spines on dorsal surface, at apex with a pair of dorsal spines and two pairs of ventral spines, intero-dorsal spine nearly equal in length to hind basitarsus. Hind basitarsus with one dorsal apical spine.

Posterior margins of all abdominal tergites straight. Epiproct semicircular; paraproct triangular in lateral view. Cercus slender, conical, apex acute. Dorsal sclerite of genitalia quadrangular, basal half rectangular, apical half wide, slightly concave; basal area of dorsal median lobe with paired lateral sclerites, oblong, apical area not divided; dorsal lateral lobes little longer than dorsal median lobe, basal area broad, gradually narrowing to apices, apical area sclerotized, hooked; ventral median lobe and ventral lateral lobes short. Subgenital plate transverse and wide, posterior margin straight.

Female: Unknown.

**Coloration.** Body light brown, with yellow spots. Eyes black. Apices of all femora with ring-like black stripes.

**Measurements (mm).** Body: ♂16.6; pronotum: ♂5.4; fore femur: ♂10.3; hind femur: ♂19.8; hind tibia: ♂20.8; hind basitarsus: ♂4.0.

**Distribution.** China (Hubei).

**Figure 24:**
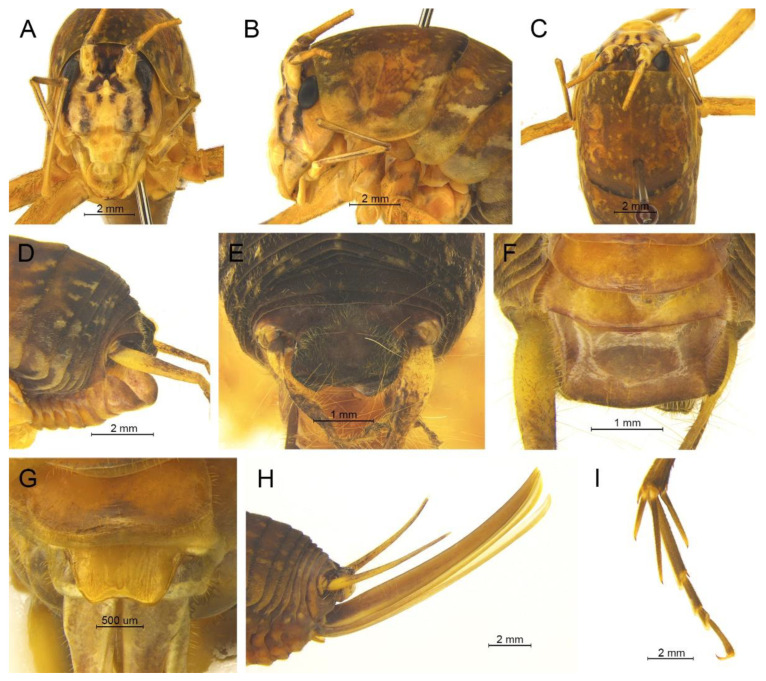
*Homotachycines fusus* Zhu & Shi sp. nov., male: (**A**–**C**) head and pronotum: (**A**) frontal view, (**B**) lateral view, (**C**) dorsal view; (**D**–**F**) apex of abdomen: (**D**) lateral view, (**E**) dorsal view, (**F**) ventral view; (**I**) hind tarsus in lateral view; female (nymph): (**G**) subgenital plate in ventral view; (**H**) ovipositor in lateral view.

***Homotachycines fusus* Zhu & Shi sp. nov.**

urn:lsid:zoobank.org:act:85F797F6-1C52-4667-9639-B740D5F53059

(Figure 20G,H and Figure 24)

**Material examined.** Holotype: ♂, China, Henan, Luoyang, Longyuwan, 8.Ⅶ.2021, coll. G.L. Hu, dep. HBU. Paratype: 1♂, same data as for holotype. Other material: 1♀ (nymph), same data as for holotype.

**Etymology.** The name of the new species derives from the Latin word ‘*fus*’ (fusiform), referring to the lateral sclerite of dorsal median lobe of the male genitalia fusiform.

**Diagnosis.** The new species is distinguished from known congeneric species by the structure of the male genitalia. The dorsal sclerite of the male genitalia is quadrate, with the basal and lateral margins slightly concave. The lateral sclerite of the dorsal median lobe of the male genitalia is fusiform.

**Description.** Male: Body medium sized in *Homotachycines*. Fastigium verticis with two conical tubercles, apices rounded, directing forward. Eyes ovoid, protruding forward; median ocellus oval, located between antennal sockets; lateral ocelli circular, situated on lateral margins of basal fastigium verticis. Apical segment of maxillary palpus much longer than subapical segment, apex inflated, globular.

Pronotum long, anterior margin straight, posterior margin arcuate; lateral lobes longer than high, ventral margins arc shaped. Mesonotum and metanotum short, posterior margin of mesonotum arcuate, posterior margin of metanotum straight. Fore coxa with one small spine; femur unarmed on ventral surface, internal genicular lobe unarmed, external genicular lobe with one long spine; tibia with two inner spines and two outer spines on ventral surface, apical area with one extero-dorsal spine and a pair of ventral spines, between the paired ventral spines with one small spine. Mid femur unarmed on ventral surface, internal and external genicular lobes each with one long spine; tibia with one inner spine and two outer spines on ventral surface, apical area with a pair of dorsal spines and a pair of ventral spines, between the paired ventral spines with one small spine. Hind femur with three to five inner spines on ventral surface, internal genicular lobe unarmed; tibia with 51–58 inner spines and 54–59 outer spines on dorsal surface, with a pair of dorsal spines in subapical area, and at apex with a pair of dorsal spines and two pairs of ventral spines, intero-dorsal spine longer than hind basitarsus. Hind basitarsus with one dorsal apical spine.

Posterior margins of all abdominal tergites straight. Epiproct semicircular; paraproct triangular in lateral view. Cercus slender, conical, apex acute. Dorsal sclerite of genitalia quadrate, basal and lateral margins slightly concave; basal area of dorsal median lobe with paired lateral sclerites, fusiform, apical area not divided; dorsal lateral lobes little longer than dorsal median lobe, basal area broad, gradually narrowing to apices, apical area sclerotized, hooked; ventral median lobe and ventral lateral lobes short. Subgenital plate transverse and wide, posterior margin straight.

Female (nymph): Appearance is similar to male. Ovipositor longer than half the length of hind femur, dorsal and ventral margins smooth. Subgenital plate trapezoid, posterior margin with a concavity.

**Coloration.** Body yellowish brown, apex of abdomen black. Face with four longitudinal black stripes, eyes black.

**Measurements (mm).** Body: ♂12.4–13.3, ♀15.4; pronotum: ♂6.0–6.4, ♀6.2; fore femur: ♂9.1–9.5, ♀8.4; hind femur: ♂18.7–19.1, ♀17.6; hind tibia: ♂19.8–20.3, ♀18.1; hind basitarsus: ♂3.5–4.1, ♀3.4; ovipositor: 13.8.

**Distribution.** China (Henan).

**Figure 25:**
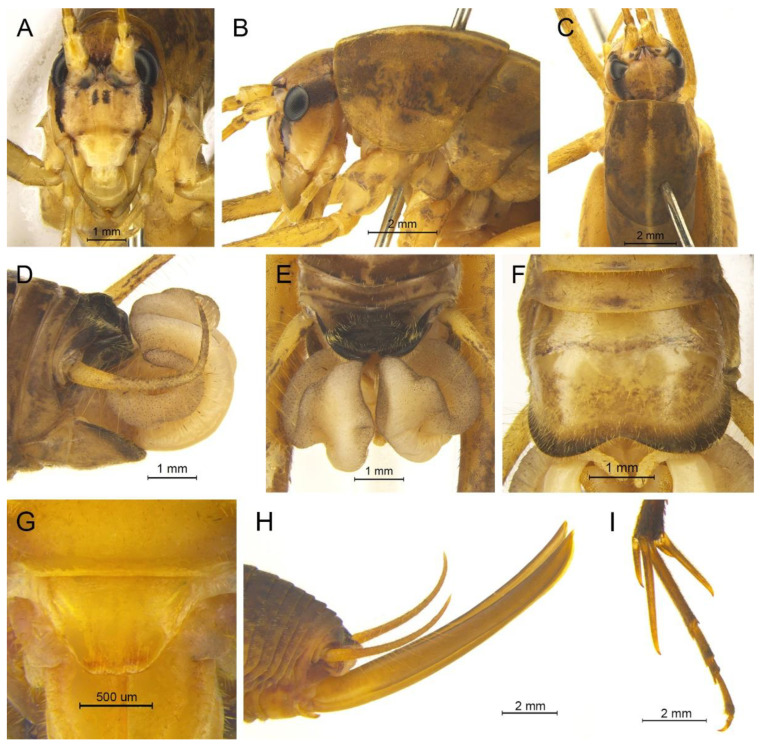
*Homotachycines concavus* Zhu & Shi sp. nov., male: (**A**–**C**) head and pronotum: (**A**) frontal view, (**B**) lateral view, (**C**) dorsal view; (**D**–**F**) apex of abdomen: (**D**) lateral view, (**E**) dorsal view, (**F**) ventral view; (**I**) hind tarsus in lateral view; female (nymph): (**G**) subgenital plate in ventral view; (**H**) ovipositor in lateral view.

***Homotachycines concavus* Zhu & Shi sp. nov.**

urn:lsid:zoobank.org:act:04A2480F-C810-4169-9B5F-4B02C2ECD16C

(Figure 20I,J and Figure 25)

**Material examined.** Holotype: ♂, China, Henan, Luoyang, Longyuwan, 8.Ⅶ.2021, coll. G.L. Hu, dep. HBU. Other material: 1♀ (nymph), same data as for holotype; 3♂, China, Shaanxi, Feng County, Hekou, 6.Ⅶ.2021, coll. J. Li, dep. HBU; 1♂, China, Shaanxi, Liuba, Zhangliangmiao, 4.Ⅷ.2017, coll. Z.X. Li, dep. HBU; 2♂, China, Shaanxi, Liuba, Zhangliangmiao, 11.Ⅶ.2021, coll. J. Li, dep. HBU; 2♂, China, Shaanxi, Mian County, Miaoping, 22.Ⅶ.2021, coll. J. Li, dep. HBU.

**Etymology.** The name of the new species is the Latin words ‘*concav*’ (concave), referring to the concave posterior margin of male subgenital plate.

**Diagnosis.** The new species can easily be distinguished from known congeneric species by the structure of the male genitalia. The basal two-thirds of the dorsal sclerite of the male genitalia are narrow and obtuse, the apical one-third is wide with the apex concave. The lateral sclerite of the dorsal median lobe of the male genitalia is nearly rectangular.

**Description.** Male: Body medium sized in *Homotachycines*. Fastigium verticis with two conical tubercles, apices rounded, directing forward. Eyes ovoid, protruding forward; median ocellus oval, located between antennal sockets; lateral ocelli circular, situated on lateral margins of basal fastigium verticis. Apical segment of maxillary palpus much longer than subapical segment, apex inflated, globular.

Pronotum long, anterior margin of disc straight, posterior margin arcuate; lateral lobes longer than high, ventral margins arc shaped. Mesonotum and metanotum short, posterior margin of mesonotum arcuate, posterior margin of metanotum straight. Fore coxa with one small spine; femur unarmed on ventral surface, internal genicular lobe with one small spine, external genicular lobe with one long spine; tibia with two inner spines and two outer spines on ventral surface, apical area with one extero-dorsal spine and a pair of ventral spines, between the paired ventral spines with one small spine. Mid femur unarmed on ventral surface, internal and external genicular lobes each with one long spine; tibia with one inner spine and one outer spine on ventral surface, apical area with a pair of dorsal spines and a pair of ventral spines, between the paired ventral spines with one small spine. Hind femur with three to eleven inner spines on ventral surface, internal genicular lobe unarmed; tibia with 60 inner spines and 54 outer spines on dorsal surface, with a pair of dorsal spines in subapical area, and at apex with a pair of dorsal spines and two pairs of ventral spines, intero-dorsal spine longer than hind basitarsus. Hind basitarsus with one dorsal apical spine.

All abdominal tergites without process. Epiproct semicircular; paraproct triangular in lateral view. Cercus slender, conical, apex acute. Basal two thirds of dorsal sclerite of genitalia narrow, obtuse, apical one third wide, apex concave; basal area of dorsal median lobe with paired lateral sclerites, nearly rectangular, apical area not divided; dorsal lateral lobes longer than dorsal median lobe, basal area broad, gradually narrowing to apices, apical area weakly sclerotized; ventral median lobe and ventral lateral lobes short. Subgenital plate transverse and wide, posterior margin concave.

Female (nymph): Appearance is similar to male. Ovipositor longer than half the length of hind femur, with dorsal and ventral margins smooth. Subgenital plate trapezoid, posterior margin straight.

**Coloration.** Body light brown, face with two longitudinal black stripes, eyes black.

**Measurements (mm).** Body: ♂15.1–15.5, ♀13.6; pronotum: ♂5.0–5.6, ♀5.3; fore femur: ♂7.4–7.7, ♀7.6; hind femur: ♂15.9–16.2, ♀15.7; hind tibia: ♂15.6–16.1, ♀15.5; hind basitarsus: ♂3.1–3.2, ♀3.2; ovipositor: 12.5.

**Distribution.** China (Henan, Shaanxi).

**Figure 26:**
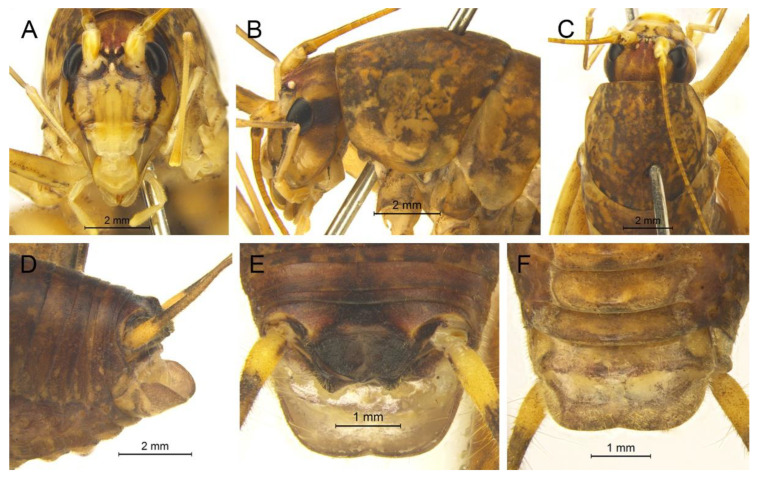
*Homotachycines qinlingensis* Zhu & Shi sp. nov., male: (**A**–**C**) head and pronotum: (**A**) frontal view, (**B**) lateral view, (**C**) dorsal view; (**D**–**F**) apex of abdomen: (**D**) lateral view, (**E**) dorsal view, (**F**) ventral view.

***Homotachycines qinlingensis* Zhu & Shi sp. nov.**

urn:lsid:zoobank.org:act:0755A42B-F2A2-4B61-92A6-01C28FE56EFA

(Figure 20K,I and Figure 26)

**Material examined.** Holotype: ♂, China, Shaanxi, Liuba, Huoshaodian, 4.Ⅹ.2020, coll. J. Li, dep. HBU. Paratype: 1♂, China, Shaanxi, Lueyang, Wulongdong, 11.Ⅹ.2020, coll. J. Li, dep. HBU. Other material: 3♂, China, Shaanxi, Yang County, Huayang town, 6.Ⅷ.2017, coll. H.Y. Liu, dep. HBU.

**Etymology.** The name of the new species derives from the type locality, which is located in the Qinling Mountains.

**Diagnosis.** The new species can be distinguished from known congeneric species by the dorsal sclerite of the male genitalia trapezoidal, the basal area with a concavity, and the apical area that is widely concave; the lateral sclerite of the dorsal median lobe of the male genitalia is oval.

**Description.** Male: Body medium sized in *Homotachycines*. Fastigium verticis with two conical tubercles, apices rounded, directing forward. Eyes ovoid, protruding forward; median ocellus oval, located between antennal sockets; lateral ocelli circular, situated on lateral margins of basal fastigium verticis. Apical segment of maxillary palpus much longer than subapical segment, apex inflated, globular.

Pronotum long, anterior margin of disc straight, posterior margin arcuate; lateral lobes longer than high, ventral margins arc shaped. Mesonotum and metanotum short, posterior margin of mesonotum arcuate, posterior margin of metanotum straight. Fore coxa with one small spine; femur with 19–25 spines on ventral surface, internal genicular lobe with one small spine, external genicular lobe with one long spine; tibia with two inner spines and two outer spines on ventral surface, apical area with one extero-dorsal spine and a pair of ventral spines, between the paired ventral spines with one small spine. Mid femur unarmed on ventral surface, internal and external genicular lobes each with one long spine; tibia with one inner spine and two outer spines on ventral surface, apical area with a pair of dorsal spines and a pair of ventral spines, between the paired ventral spines with one small spine. Hind femur with two to four inner spines on ventral surface, internal genicular lobe with one small spine; tibia with 53–57 inner spines and 55–59 outer spines on dorsal surface, with a pair of dorsal spines in subapical area, and at apex with a pair of dorsal spines and two pairs of ventral spines, intero-dorsal spine slightly longer than hind basitarsus. Hind basitarsus with one dorsal apical spine.

All abdominal tergites without process. Epiproct semicircular; paraproct triangular in lateral view. Cercus slender, conical, apex acute. Dorsal sclerite of genitalia trapezoidal, basal area with a concavity, apical area widely concave; basal area of dorsal median lobe with paired lateral sclerites, oval, apical area not divided; dorsal lateral lobes longer than dorsal median lobe, basal area broad, gradually narrowing to apices, apical area sclerotized, apex acute; ventral median lobe and ventral lateral lobes short, cylindrical. Subgenital plate transverse and wide, posterior margin slightly concave.

Female: Unknown.

**Coloration.** Body light brown. Face with two longitudinal black stripes, eyes black, ocelli yellow. All femora with ring black stripes, basal half of hind femur with pinnate black stripes.

**Measurements (mm).** Body: ♂14.3–16.6; pronotum: ♂5.3–5.4; fore femur: ♂7.4–7.5; hind femur: ♂15.2–16.5; hind tibia: ♂14.8–17.2; hind basitarsus: ♂3.1–3.2.

**Distribution.** China (Shaanxi).

## 4. Discussion

### 4.1. Phylogenetic Analyses

According to the current classification of *Gymnaetoides* and *Pseudotachycines*, the biggest difference between them is the distinctly prolonged male paraproct in *Pseudotachycines* [13,14]. However, the result of phylogenetic analyses shows that both *Gymnaetoides* and *Pseudotachycines* are paraphyletic, which is inconsistent with morphological classification. Therefore, we examine the type specimens of all known species and check the morphological characters of all species. In addition to the shape of the male paraproct, there are distinct differences in the shape of the male genitalia, especially in the shape of the dorsal lateral lobes and the dorsal median lobe of it. Based on the morphological characters and molecular data, we revise the genera *Gymnaetoides* and *Pseudotachycines* and a new genus *Homotachycines* Zhu & Shi gen. nov. is erected.

*Gymnaetoides*. It can easily be distinguished from *Pseudotachycines* and *Homotachycines* by the apical area of the dorsal median lobe of the male genitalia being divided into two lobes, and the dorsal lateral lobes of the male genitalia are of nearly equal length as the dorsal median lobe.

*Pseudotachycines*. The genus is similar to *Homotachycines*, but it differs from the latter one by the prolonged male paraproct and the much longer dorsal lateral lobes of the male genitalia, in which the apical area is almost as wide as the basal area.

*Homotachycines*. It is similar to *Pseudotachycines*, but it can be distinguished by the normally developed male epiproct and paraproct and the slightly longer dorsal lateral lobes of the male genitalia, in which the apical area is much narrower than the basal area.

*Homotachycines* is sister to *Pseudotachycines*, and they are together sisters to *Gymnaetoides*. *Homotachycines* and *Pseudotachycines* are similar in appearance to some degree. However, the dorsal lateral lobes of the male genitalia between them are distinctly different. Moreover, considering that the genus *Megatachycines* has developed male epiproct (quadrangular or pentagonal, much longer than the paraproct) [15], we believe that the specialized male epiproct or paraproct may be important identifying characters. Therefore, we consider *Homotachycines* and *Pseudotachycines* as two separate genera.

According to the revised generic diagnoses, *P. deformis*, *P. inermis*, and *P. yueyangensis* of the genus *Pseudotachycines* should be transferred to *Gymnaetoides*. Moreover, *P. ovalilobatus* and *P. trapezialis* of the genus *Pseudotachycines* and *G. acutilobatus* of the genus *Gymnaetoides* should be transferred to the genus *Homotachycines*. As a consequence, six new taxonomic combinations are proposed: *Gymnaetoides deformus* comb. nov., *G. inermus* comb. nov., *G. yueyangensis* comb. nov., *Homotachycines acutilobatus* comb. nov., *H. ovalilobatus* comb. nov., and *H. trapezialis* comb. nov.

Last but not least, we find that the number of spines on hind femur is neither stable between different congeners nor between different individuals of the same species. Therefore, we do not think that the arrangement of spines could be a “good character” for identifying the genera, and we support Gorochov’s view that the structures of the male genitalia are the more important characters [46]. As mentioned above, the classifications of genera need to be supported by more evidence and more valid characters need to be discovered.

### 4.2. Integrative Taxonomy

The results of the four species delimitation methods are inconsistent. The ABGD and jMOTU analyses result in 22 MOTUs, which are completely consistent with morphological species. In contrast, the bPTP and GMYC analyses generate more MOTUs. Consequently, we examine all the specimens in which results of four species delimitation methods were inconsistent. However, no distinct difference is found between different MOTUs. The more MOTUs generated by the PTP and GMYC methods might be due to the high rates of false positives of them [47,48,49,50,51].

There is also inconsistency between molecular data and morphological species. In *Pseudotachycines procerus*, specimens from Guizhou and Anhui form two MOTUs in all the four species delimitation methods. However, based on the morphological characters, there is only slightly difference in the lateral sclerite of the dorsal median lobe of the male genitalia, and that is not distinct enough to separate them into two independent species. Therefore, we consider specimens from Guizhou as a subspecies.

As mentioned above, 21 species and one subspecies are identified based on morphological characters and molecular evidence, of which 15 species and one subspecies are newly described: *Gymnaetoides huangshanensis* Zhu & Shi sp. nov., *G. petalus* Zhu & Shi sp. nov., *G. yangmingensis* Zhu & Shi sp. nov., *G. lushanensis* Zhu & Shi sp. nov., *Pseudotachycines procerus* Zhu & Shi sp. nov., *P. procerus guizhouensis* Zhu & Shi ssp. nov., *P. zhengi* Zhu & Shi sp. nov., *P. nephrus* Zhu & Shi sp. nov., *P. sagittus* Zhu & Shi sp. nov., *P. fengyangshanensis* Zhu & Shi sp. nov., *Homotachycines triangulus* Zhu & Shi sp. nov., *H. quadratus* Zhu & Shi sp. nov., *H. baokangensis* Zhu & Shi sp. nov., *H. fusus* Zhu & Shi sp. nov., *H. concavus* Zhu & Shi sp. nov., and *H. qinlingensis* Zhu & Shi sp. nov.

The results indicate that the molecular species delimitation methods can successfully identify most species in these genera. However, the inconsistency of morphological and genetic data or about that of different species delimitation methods suggest that species delimitation should implement multiple methods and conclusions should be drawn by integrating morphological characters and molecular data [50]. Moreover, we verify that the shapes of the dorsal and lateral sclerites of the male genitalia are suitable for the classifications of species.

To sum up, the structures of the genitalia are crucial for species diagnosis and generic identification for the three genera. In the tribe Aemodogryllini, the genitalia have sclerotized structure, while those of the tribe Diestramimini are membranous except for the genus *Megadiestramima* [46,52]. Therefore, Aemodogryllini is considered as a younger tribe, diverging from Diestramimini [46]. Moreover, the mating behavior of Rhaphidophoridae is unique, which may have played an important role in the evolutionary history of Orthoptera. Reproductive isolation presumable results in speciation [53,54,55,56]. The evolution of the genitalia and consequence on speciation are interesting scientific questions that can be further studied in the future.

## 5. Conclusions

Phylogenetic analyses suggest that both genera *Gymnaetoides* and *Pseudotachycines* are paraphyletic. Therefore, we revise their taxonomy based on the morphological characters and molecular data. A new genus is established, and six new taxonomic combinations are proposed. Species delimitation identifies 15 new species and one new subspecies. Moreover, we find that the shapes of the dorsal lateral lobes and the dorsal median lobe of the male genitalia are also important characters for identifying these genera, and that the shapes of the dorsal and lateral sclerites of the male genitalia are suitable for the classifications of species.

## Data Availability

The data that support the findings of this study are openly available in National Center for Biotechnology Information, accession numbers ON129823–ON129878, ON146600–ON146820.

## Supplementary Materials

The following supporting information can be downloaded at: https://www.mdpi.com/article/10.3390/insects13070628/s1, Table S1: Information on the specimens included in this study and related GenBank accession numbers.
